# Quantifying the association between pregnancy exposure to biomass-attributable PM_2.5_ and the risk of preterm birth and stillbirth: A case-control study in Sydney, Australia for 2010–2020

**DOI:** 10.1097/EE9.0000000000000381

**Published:** 2025-03-26

**Authors:** Tanya Singh, Joe Van Buskirk, Geoffrey Morgan, Katrin Meissner, Donna Green, Edward Jegasothy

**Affiliations:** a Climate Change Research Centre, University of New South Wales, Sydney, New South Wales, Australia; b ARC Centre of Excellence for Climate Extremes, University of New South Wales, Sydney, New South Wales, Australia; c Sydney School of Public Health, The University of Sydney, Sydney, New South Wales, Australia; d Faculty of Medicine and Health, The University of Sydney, Sydney, New South Wales, Australia; e Health Environments and Lives (HEAL) National Research Network, Australia; f Centre for Safe Air, NHMRC Centre for Research Excellence, Hobart, Tasmania, Australia; g School of Biological, Earth and Environmental Sciences, University of New South Wales, Kensington, Sydney, NSW, Australia

**Keywords:** PM_2.5_, Biomass combustion, Biomass-attributable PM_2.5_, Wildfires, Wood heaters, Preterm birth, Stillbirth, Case–control design

## Abstract

Biomass combustion, including wildfires and residential wood burning, is a significant source of particulate matter (PM_2.5_) in Australia, with potentially distinct health effects due to its unique chemical composition. This study aimed to quantify the association between exposure to ambient biomass-attributable PM_2.5_ and the risk of preterm birth and stillbirth across pregnancy windows in Sydney, Australia, from 2010 to 2020. We conducted case–control studies nested within a cohort of 578,391 singleton pregnancies, including 29,954 preterm births and 2,928 stillbirths. Controls were randomly selected using risk-set sampling. Daily all-source PM_2.5_ estimates at a 5 km resolution were obtained from a previous study. Days exceeding the 95th percentile of all-source PM_2.5_ at statistical area level 4 without significant dust storm pollution were classified as biomass-affected days. For these days, biomass-attributable PM_2.5_ was estimated using the remainder component from a seasonal trend decomposition, with the seasonal and trend components representing nonbiomass-attributable PM_2.5_. Conditional logistic regressions were used to analyze associations between biomass-attributable PM_2.5_ exposure and outcomes, adjusting for area-level socioeconomic factors, temperature, humidity, and temporal and seasonal trends. The odds ratio for preterm birth per interquartile range increase in biomass-attributable PM_2.5_ was 1.002 (95% CI = 0.997, 1.007) for the entire pregnancy average exposure, with similar null results across trimesters. For stillbirth, the odds ratio was 1.002 (95% CI = 0.985, 1.019) for the entire pregnancy average exposure, with comparable null findings across trimesters. These results suggest that in Sydney, biomass-attributable PM_2.5_ exposure during pregnancy may not increase the risk of preterm births or stillbirths.

**What this study adds**

This study provides one of the first analyses of biomass-attributable particulate matter (PM_2.5_) exposure on preterm births and stillbirths in a low air pollution setting with episodic extreme fire events. Applying an established decomposition approach to identify biomass-attributable PM_2.5_, we examined pregnancy and trimester-specific exposure patterns in Sydney, Australia. Despite hypotheses about enhanced toxicity of biomass-related PM_2.5_, we found no clear associations with adverse birth outcomes when using average exposure metrics. These findings contribute important evidence to the limited and mixed literature on perinatal risks from biomass smoke exposure during pregnancy.

## Introduction

Fine particulate matter (PM_2.5_), an air pollutant particle of 2.5 micrometers or smaller in diameter, has been linked to numerous health issues,^1,2^ including adverse birth outcomes such as preterm births^3^ and stillbirths.^4^ Biomass burning from wood combustion, power generation, and landscape fires significantly contributes to PM_2.5_, particularly in Canada, Siberia, Africa, South America, and Australia.^5–9^ Landscape fires can encompass both controlled and uncontrolled vegetative fires (i.e., wildfires) in natural areas like forests, grasslands, bushlands, and shrublands.

Literature suggests that the composition of ambient PM_2.5_ from biomass combustion (i.e., biomass-specific PM_2.5_) differs from other sources, potentially leading to distinct health effects.^5,10–16^ The biological plausibility of these findings is supported by toxicological studies showing that biomass-specific PM_2.5_, with its higher proportions of carbonaceous material and more polar organic compounds, leads to enhanced oxidative stress and inflammation compared with urban nonbiomass-related ambient PM_2.5._^6,12^ A systematic review found significant associations between exposure to PM_2.5_ from biomass burning and increased risk of all-cause mortality, respiratory morbidity, and, to a lesser extent, cardiovascular morbidity.^14^

Recent unprecedented wildfires, such as the 2019–2020 Australian wildfires and the 2020 Western United States wildfires, have raised concerns about the impact of landscape fire smoke—a major contributor to biomass smoke^9^—on vulnerable populations, including pregnant women and their infants.^17–19^ The first systematic review on this topic, published in 2021, found limited evidence on the association between landscape fire exposure during pregnancy and adverse pregnancy outcomes, with mixed evidence for preterm birth and insufficient evidence for stillbirths.^17^ Most studies published after this first review have found a positive association between landscape fire and preterm births.^18–23^ Evidence on the association between landscape fire exposure and stillbirths, however, remains limited and conflicting. A recent multicity study from New South Wales (NSW), Australia, focusing on short-term wildfire-specific PM_2.5_ exposure before delivery, reported a hazard ratio of 1.40 (95% confidence interval [CI] = 1.11, 1.78) for stillbirth over a lag of 0–6 days,^24^ while another study focusing exclusively on the extreme wildfires during 2019–2020 in Sydney, Australia, found no significant positive association.^25^ These conflicting results highlight the need for further research to clarify the relationship between landscape fire exposure and adverse pregnancy outcomes, particularly stillbirths.

Pollution from biomass-specific PM_2.5_, particularly from landscape fires and residential wood heaters, contributes substantially to episodes of extreme PM_2.5_ pollution in Australia, a country that otherwise experiences good air quality with an annual average PM_2.5_ level of approximately 8 μg/m^3^.^8,26–28^ The potential harms associated with ambient biomass-specific PM_2.5_ have recently received increased attention nationally.^8,11,29^ While studies examining the effect of the more commonly studied exposure of PM_2.5_ from all sources (i.e., all-source PM_2.5_) on preterm births and stillbirths in Australia have produced inconsistent results,^30–33^ the effects of biomass-specific PM_2.5_ may differ from those of nonbiomass PM_2.5_ sources.^5,10–16^ From a public health perspective, it is important to understand the specific health risks posed by biomass-specific PM_2.5_ on perinatal outcomes, especially as landscape fires are projected to increase in frequency and severity due to climate change.^11^

In this study, we aimed to quantify the association between pregnancy exposure to biomass-attributable PM_2.5_, a proxy for biomass-specific PM_2.5,_ and the risk of preterm birth and stillbirth in Sydney, Australia, from 2010 to 2020. By assessing both overall pregnancy and trimester-specific exposure, we sought to capture typical PM_2.5_ levels experienced by pregnant women in Sydney and identify potential windows of vulnerability during pregnancy. Given that pregnant women in Sydney are exposed to smoke from both landscape fires and wood heaters—particularly during winter—it can be challenging to attribute potential risks solely to landscape fire events. These two sources of biomass combustion, despite occurring at different times and for different reasons, share many common pollutants. Thus, considering the combined exposure from both sources provides a more comprehensive understanding of the risks. We also compared these findings with exposure to all-source PM_2.5_ to investigate whether distinct types of PM_2.5_, such as biomass-attributable versus all-source, have different effects.

## Methods

We conducted case–control studies for each combination of exposures and outcomes for mothers residing in metropolitan Sydney. Sydney, the most populous city in Australia, with a population of 5.2 million in 2021^34^ was severely affected by the 2019–2020 wildfires, with 81 days of poor or hazardous air quality in 2019, more than the previous 10 years combined.^35^

### Data

#### Birth data/study population

Before applying any exclusion criteria and creating case–control risk sets, our initial study population comprised all singleton births conceived between 28 November 2009 and 03 February 2020 (N = 583,353) from mothers residing in metropolitan Sydney. Data for these births were retrieved from the NSW Perinatal Data Collection. It contains all births reported in public and private hospitals, and home births within NSW.^36^ Relevant attributes for this study include: birth date, gestational age at birth (in completed weeks, based on the best clinical estimate), parity (1, > 1), maternal age at delivery (in years), smoking status of the mother during pregnancy (ever smoked vs. never smoked), perinatal death (not a perinatal death, stillborn, other deaths) and the residential location of the mother at the time of birth, represented by their statistical area level 2 (SA2). SA2 is a spatial unit, part of the Australian Statistical Geography Standard 2011, which provides hierarchical spatial divisions for the classification of data. Our study area comprises 236 SA2s with a median area of 8.0 km² (5th percentile = 2.5 km², 95th percentile = 81.6 km²). Furthermore, we obtained the Index of Relative Socioeconomic Disadvantage (IRSD), one of the four SocioEconomic Indexes for Areas produced by the Australian Bureau of Statistics.^37^ IRSD ranks areas based on a weighted sum of census-derived population characteristics associated with disadvantage, including indicators of income, education, employment, housing, and English language proficiency. Sydney SA2s were ranked into quintiles based on their IRSD scores, with lower scores indicating relatively greater disadvantage. While IRSD is a widely used measure of disadvantage, it characterizes areas rather than representing the socioeconomic status of an individual.

We restricted our case–control study to women with complete maternal information and to pregnancies with conception dates between 20 weeks before the birth cohort’s start and 43 weeks before the cohort’s end, avoiding fixed cohort bias (see Figure S1; http://links.lww.com/EE/A335 for the different exclusion criteria applied).^38,39^ These criteria resulted in 572,681 (98.2%) pregnancies eligible for the stillbirth analysis. For the preterm birth analysis, we further limited our cohort to live births (564,693; 96.8%). From these cohorts, we nested a case–control study, identifying all stillbirth (N = 2928; 0.5%) and preterm birth cases (N = 29,954; 5.3%). We employed a risk-set sampling approach with replacement to select controls to best represent the underlying cohort, minimize selection bias, and control for time-varying exposures while ensuring comparable exposure time windows through gestational age matching.^40,41^ For each preterm birth case, we randomly selected 10 eligible controls still in utero at the corresponding gestational age (N = 299,540). For stillbirths, we applied a similar method for selecting controls by not only considering infants still in utero but also live births at the same gestational age (N = 29,280). This process yielded a total of 329,494 births for the preterm and 32,208 for the stillbirth analysis.

#### Exposure data

##### Air pollution

Ambient air pollution exposure data was provided by the “The Clean Air and Health Research Data and Analysis Technology (CARDAT) (Bushfire Smoke modeling project version 1.3),”^42^ the Centre for Safe Air’s data sharing platform. The underlying air pollution model estimated daily concentrations of PM_2.5_ at a 5 km grid cell level for Australia from 2001 to 2020, using PM_2.5_ data from air pollution monitoring sites, combined with satellite, weather, and land use data using a random forest statistical model, which achieved a final out‐of‐bag R² of around 71.5% and a root-mean-square error (RMSE) of 4.5 μg/m^3^. The model performance was comparable to other international experiences. Then a Seasonal Trend Decomposition using the LOESS (STL) algorithm was applied to estimate the season and trend components of the PM_2.5_ time series.^43^ This method divides the air pollution time series into its three components: trend (longer-term tendency), seasonal (repeated patterns), and remainder (deviations from the seasonal and trend components). Summing these three components provides the all-source PM_2.5_ concentration for a given day and location. The resulting estimates were then aggregated to the SA2 level for our analysis to match the spatial resolution of our birth data.

To identify days with potential biomass-attributable PM_2.5_ pollution, we first determined extreme air pollution days for the period from 28th November 2009, to 30th June 2020, adapting a similar method previously applied.^44^ While PM_2.5_ data was initially available at the SA2 level, we conducted this step at the statistical area 4 (SA4) level because landscape fire smoke typically affects broader geographical areas uniformly. For each of Sydney’s 14 SA4, which are the largest sub-state regions in the Australian Statistical Geography Standard, we aggregated estimates from SA2 level to SA4 and calculated simple means. An extreme air pollution day was then defined as one where this SA4-level mean exceeded the 95th percentile of all-source PM_2.5_ for that SA4. We then excluded dust-affected days to identify biomass-attributable PM_2.5_ days. Dust-affected days were identified in the CARDAT dataset using two sources: the Modern-Era Retrospective Analysis for Research and Applications, version 2 (MERRA-2), and the Copernicus Atmosphere Monitoring Service (CAMS) reanalysis data. A day was classified as dust-affected if the 75th percentile threshold was surpassed for either dust storm indicator on that day. These thresholds were established based on Australia-wide data, covering the period from 1st January 2001 to 30th June 2020. Once a day was classified as having biomass-attributable PM_2.5_, the remainder component from the STL was used to estimate biomass-attributable PM_2.5_ on an SA2 level. On such days the seasonal and trend components represented nonbiomass-attributable PM_2.5_ and all-source PM_2.5_ was the sum of nonbiomass-attributable PM_2.5_ and biomass-attributable PM_2.5_.

##### Meteorological data

Daily average temperature (°C) and relative humidity, with a 0.05° × 0.05° resolution were obtained from the Australian Gridded Climate Data (v1.0.0) from the Australian Bureau of Meteorology and an area-weighted average was calculated per day for each SA2.^45^

##### Exposure assessment

Exposure assessment was conducted at the area level, with mothers assigned daily biomass-attributable PM_2.5_, nonbiomass-attributable PM_2.5_, all-source PM_2.5_, ambient temperature, and relative humidity values based on their residential SA2. This area-level approach is consistent with other environmental epidemiology studies examining population-level associations between air pollution and birth outcomes. To ensure valid exposure comparison, we assigned gestational age-adjusted exposure levels to matched controls based on the gestational length of the case, regardless of the gestational lengths of the controls. This approach is essential for unbiased comparison as it ensures that exposure periods are identical between cases and controls, similar to time-to-event analyses.^40,41,46^ For each matched set, we calculated average gestational age-adjusted exposure based on the truncated gestational length of controls for biomass-attributable PM_2.5_, nonbiomass-attributable PM_2.5_, all-source PM_2.5,_ temperature, and relative humidity from daily exposure data. Trimester-specific average exposures were defined as following: 0–13 completed weeks as the 1st trimester, 14–27 completed weeks as the 2nd trimester, and after 27 completed weeks as the 3rd trimester.^47^

#### Outcomes

We define preterm births as any live birth before 37 completed gestational weeks.^48^ In Australia, a stillbirth is defined as a fetal death before the birth of an infant of at least 20 completed weeks of gestation or 400 grams birthweight and is flagged as such in the Perinatal Data Collection.^49^

#### Covariates

Using a directed acyclic graph (DAG) informed by literature, we identified the minimal sufficient set of confounders—variables that are associated with both biomass-attributable PM_2.5_ exposure and birth outcomes, which when controlled for, block all backdoor paths between exposure and outcome while avoiding unnecessary adjustment (Figure S2; http://links.lww.com/EE/A335).^50^ In our primary analysis we adjusted for these potential confounders: area-level IRSD, natural cubic spline for ambient relative humidity and temperature with three degrees of freedom (df), natural cubic spline for the year of conception with 2 df, and an indicator variable for the month of conception. The latter two variables were added to adjust for long-term trends and seasonality, respectively. We note that, while some of the variables presented in the DAG like maternal age, smoking status, body mass index, or maternal age, are associated with socioeconomic status, they are indirect proxies that may act through different causal pathways to influence birth outcomes. Area-level IRSD, in contrast, directly captures multiple dimensions of socioeconomic disadvantage that could confound the relationship between residential PM_2.5_ exposure and birth outcomes.

### Statistical analyses

To examine relationships among the exposure variables, we calculated Spearman correlation coefficients for the entire pregnancy and trimester-specific exposures. We employed a conditional logistical regression to analyze the association between biomass-attributable PM_2.5_ and preterm birth and stillbirth. Odds ratios (OR) and 95% confidence intervals (CI) for interquartile range (IQR) increases in biomass-attributable PM_2.5_ were calculated based on distributions within each dataset for all pregnancy windows. We tested whether the functional form for biomass-attributable PM_2.5_ was linear or nonlinear (i.e., natural cubic spline with 3 df) by calculating both the Akaike and Bayesian information criteria and by examining the exposure–response curves. Entire pregnancy exposure and trimester-specific associations were each assessed in separate models, resulting in eight different models for this study. Notably, deliveries before 28 gestational weeks were excluded from the third-trimester analyses. We chose to examine entire pregnancy and trimester-specific average exposures to understand typical exposure patterns pregnant women experience, rather than focusing on short-term exposure effects. This approach aligns with previous studies examining associations between air pollution and birth outcomes.^20,23^

To test the robustness of our results, we conducted several sensitivity analyses. While our primary analysis focused on variables identified as true confounders through our DAG, we conducted additional analyses adjusting for variables that might be associated with birth outcomes, but not necessarily with exposure: maternal age (modeled as a natural cubic spline with 3 degrees of freedom), parity, infant sex, maternal smoking status, and nonbiomass-attributable PM_2.5_. This analysis allowed us to assess whether our findings were robust to different modeling choices and to compare with previous studies that included these variables. Furthermore, our model for biomass-attributable PM_2.5_ relied on a decomposition approach, which may have underestimated seasonal biomass sources. Therefore, we performed a sensitivity analysis in which we only removed the trend component from the daily predicted PM_2.5_ concentrations and retained the seasonal component. Additionally, we conducted a sensitivity analysis restricting our analysis to births before November 2019, thereby excluding births potentially affected by two major events: the unprecedented 2019–2020 wildfires that began in November 2019 in our study area and resulted in extreme PM_2.5_ levels until February 2020,^44^ and the COVID-19 lockdown measures implemented in March 2020. The lockdown could have modified both exposure patterns (through changes in activity patterns) and psychosocial factors that may influence birth outcomes. This allowed us to examine whether our findings were driven by these exceptional circumstances or were consistent with patterns during more typical exposure periods. Finally, we employed a time-varying Cox proportional hazard model for entire pregnancy exposure to test whether an alternative statistical method yields similar results.

All analyses were performed in R (version 4.4.0; R Development Core Team), with the “survival”^51^ and “splines”^52^ packages.

This study was approved by the University of NSW Human Research Low Risk Ethics Advisory Committee Panel E, Reference Number: HC200817.

## Results

**Figure 1.**
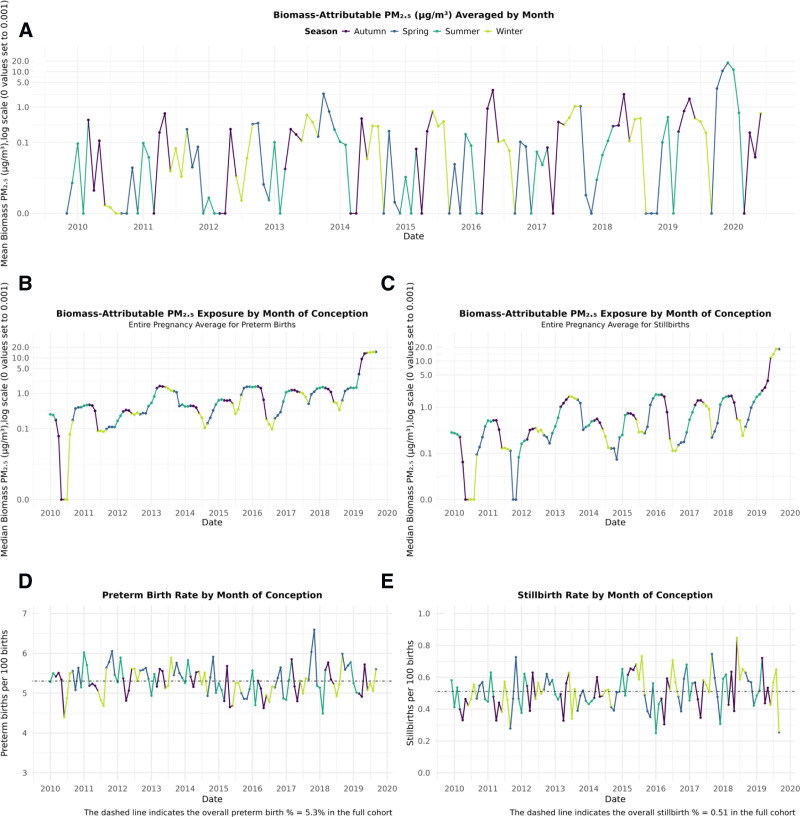
Biomass-attributable PM_2.5_ exposure and birth outcomes in metropolitan Sydney for births conceived between 04 January 2010–03 September 2019 (preterm birth set) and 05 December 2009–03 September 2019 (stillbirth set). A, Monthly time series data of biomass-attributable PM_2.5_ levels on a log scale, averaged from daily mean measurements across the entire metropolitan area. B and C, Seasonal trends in entire pregnancy exposures by month of conception for preterm births and stillbirths on a log scale, respectively. D and E, Corresponding preterm birth rates and stillbirth rates by month of conception.

The study population included 33,208 singleton deliveries for the stillbirth analysis and 329,494 singleton live births for the preterm birth analysis. As for each case, 10 controls were chosen; both case–control sets had 9.1% cases. In the stillbirth set, a total of 87.2% (N = 28,075) of pregnancies were exposed to at least 1 day of biomass-attributable PM_2.5_, of which 9.2% were stillbirths. In the preterm birth set, 92.5% (N = 305,986) of pregnancies were exposed to at least one biomass-attributable PM_2.5_ day, of which 9.1% were preterm birth cases. The high prevalence of exposure to biomass-attributable PM_2.5_ reflects our exposure assessment methodology integrating episodic and seasonal biomass sources across gestational periods, with residential wood heating contributing during winter months and landscape fires occurring predominantly in spring and summer. Landscape fires include controlled burns to reduce the risk of uncontrolled wildfires and are routinely used in Australia and in NSW to reduce wildfire risk.^29^ Figure 1 illustrates this temporal heterogeneity in biomass-attributable PM_2.5_ concentrations, with Panel A demonstrating distinct seasonal patterns: peaks typically during spring (September–November) and autumn (March–May), while winter months (June–August) show more variable elevation patterns. Panels B and C show seasonal trends in entire pregnancy exposures by month of conception on a log scale. While notable peaks in biomass-attributable PM_2.5_ exposure were observed in 2013, and 2016–2018, the most striking peak occurred during the 2019–2020 wildfires. However, this extreme event, while unprecedented in magnitude, affected only a small proportion of our study population, with most births in our analysis occurring during periods of more typical exposure levels. Throughout most of the study period, biomass-attributable PM_2.5_ levels made up only a small proportion of all-source PM_2.5_ (Figure S3; http://links.lww.com/EE/A335). Visual inspection suggests no apparent unusual increase in preterm birth and stillbirth rates corresponding to periods of higher biomass-attributable PM_2.5_ exposure (Figure 1D,E), though it is important to note that increases in risk are not necessarily detectable at this scale. The spatial distribution of median all-source PM_2.5_ and biomass-specific PM_2.5_ exposure levels across different pregnancy windows, aggregated by SA2 are shown in Figure S4; http://links.lww.com/EE/A335 with exposure hotspots concentrated around central Sydney, likely due to higher population density.

Compared to their controls, mothers of stillbirths and preterm births showed distinct characteristics (Table 1). They were more likely to be either older (>34 years) or younger (<20 years) and had a higher probability of having a male infant (particularly for preterm births). These mothers were also more frequently smokers, first-time pregnant, and living in socially disadvantaged areas. Stillbirths had a lower median gestational age compared with their controls, attributable to a significant proportion of stillbirths occurring before the onset of the third trimester (Table S1 and S2; http://links.lww.com/EE/A335).

**Table 1.** Baseline characteristics of pregnant women residing in metropolitan Sydney for preterm birth and stillbirth case–control sets and respective full cohorts

		Preterm birth matched set^a^			Stillbirth matched set^b^		
		Counts and percentages			Counts and percentages		
Characteristics		Control births^c^ (N = 299,540)	Preterm births (N = 29,954)	All live births-full cohort (N = 564,693)	Control births^d^ (N = 29,280)	Stillbirths (N = 2,928)	All births-full cohort (N = 572,681)
Median gestational age (IQR)		39.00 (2.00)	35.00 (2.00)	39.00 (2.00)	39.00 (2.00)	25.00 (13.00)	39.00 (2.00)
Mean mothers’ age (SD)		31.31 (5.17)	31.59 (5.61)	31.32 (5.19)	31.30 (5.18)	31.51 (5.58)	31.32 (5.19)
Mothers’ age group	<20	4129 (1.4%)	587 (2.0%)	7855 (1.4%)	411 (1.4%)	57 (1.9%)	8014 (1.4%)
	20–34	213,427 (71.3%)	20,136 (67.2%)	401,654 (71.1%)	20,789 (71.0%)	1981 (67.7%)	407,245 (71.1%)
	>34	81,984 (27.4%)	9231 (30.8%)	155,184 (27.5%)	8080 (27.6%)	890 (30.4%)	157,422 (27.5%)
Previous pregnancy	No	136,386 (45.5%)	15,206 (50.8%)	258,570 (45.8%)	13,579 (46.4%)	1453 (49.6%)	262,411 (45.8%)
	Yes	163,154 (54.5%)	14,748 (49.2%)	306,123 (54.2%)	15,701 (53.6%)	1475 (50.4%)	310,270 (54.2%)
Sex of infant	Boy	153,732 (51.3%)	16,509 (55.1%)	290,785 (51.5%)	15,153 (51.8%)	1527 (52.2%)	294,871 (51.5%)
	Girl	145,808 (48.7%)	13,445 (44.9%)	273,908 (48.5%)	14,127 (48.2%)	1401 (47.8%)	277,810 (48.5%)
Mothers’ smoking status during pregnancy	Nonsmoking	282,635 (94.4%)	27,028 (90.2%)	532,091 (94.2%)	27,605 (94.3%)	2672 (91.3%)	539,415 (94.2%)
	Smoking	16,905 (5.6%)	2926 (9.8%)	32,602 (5.8%)	1675 (5.7%)	256 (8.7%)	33,266 (5.8%)
Index of Relative Socioeconomic Disadvantage (IRSD) in quintiles	Most disadvantaged	59,376 (19.8%)	6674 (22.3%)	113,014 (20.0%)	5790 (19.8%)	752 (25.7%)	114,151 (20.0%)
	Second	61,267 (20.5%)	6577 (22.0%)	115,444 (20.4%)	6025 (20.6%)	653 (22.3%)	116,588 (20.5%)
	Middle	58,800 (19.6%)	5898 (19.7%)	111,249 (19.7%)	5734 (19.6%)	546 (18.6%)	112,350 (19.7%)
	Fourth	60,007 (20.0%)	5639 (18.8%)	113,113 (20.0%)	5897 (20.1%)	517 (17.7%)	114,368 (20.1%)
	Least disadvantaged	60,090 (20.1%)	5166 (17.2%)	111,873 (19.8%)	5834 (19.9%)	460 (15.7%)	112,296 (19.7%)

a For births conceived between 04 January 2010–03 September 2019.

b For births conceived between 05 December 2009–03 September 2019.

c Controls for the preterm birth set were selected from births with gestational age > gestational age of case.

d Controls for the stillbirth set were selected from ongoing pregnancies with gestational age > gestational age of case and live births of at least the same gestational age as a case.

Exposure patterns between cases and controls showed minimal differences in median values for all-source PM_2.5_, biomass-attributable PM_2.5_, and number of biomass exposure days throughout pregnancies and within trimesters (Table 2 and Table S3; http://links.lww.com/EE/A335). However, small differences emerged at extreme values (95th and 99th percentile), with cases generally showing slightly higher values for both preterm births and stillbirths. Excluding the 2019–2020 wildfire period Table S4; http://links.lww.com/EE/A335 showed substantial reductions in the maximum and extremely high values for biomass-attributable PM_2.5_, while median values showed less pronounced changes. This indicates that the 2019–2020 extreme exposure event significantly impacted the upper range of biomass-attributable PM_2.5_ levels and the number of days with biomass pollution present while having no pronounced effects on the overall exposure distribution in our cohort samples.

**Table 2.** Summary statistics of exposure variables for preterm birth and stillbirth matched sets during different pregnancy windows for pregnant women residing in metropolitan Sydney

Exposure and pregnancy window	Preterm birth set^a^	Minimum	5th	25th	Median	75th	95th	99th	Maximum	Stillbirth set^b^	Minimum	5th	25th	Median	75th	95th	99th	Maximum
All-source PM_2.5_ entire pregnancy	Control	5.13	6.16	6.95	7.57	8.13	9.26	14.04	21.08	Control	5.04	6.08	6.91	7.51	8.11	9.23	14.00	25.86
	Preterm birth	5.29	6.16	6.93	7.57	8.14	9.29	14.13	19.05	Stillbirth	5.10	6.12	6.99	7.56	8.17	9.33	14.49	19.14
Biomass-specific PM_2.5_ entire pregnancy	Control	0.00	0.00	0.07	0.19	0.44	1.13	5.91	12.86	Control	0.00	0.00	0.05	0.15	0.42	1.01	5.92	17.14
	Preterm birth	0.00	0.00	0.07	0.18	0.44	1.13	5.98	10.58	Stillbirth	0.00	0.00	0.05	0.16	0.43	1.06	6.36	10.50

a Controls are any births with gestational age > gestational age of case. For births conceived between 04 January 2010–03 September 2019.

b Controls are any ongoing pregnancies with gestational age > gestational age of case, or live births of at least the same gestational age as a case. For births conceived between 05 December 2009–03 September 2019.

The Spearman correlation analysis revealed low to moderate correlations (<|0.31|) between the exposure variables across all pregnancy exposure windows and outcome-specific models (Tables S5–S12; http://links.lww.com/EE/A335). Correlations between all-source PM_2.5_ and biomass-attributable PM_2.5_ were high (0.64–0.80), which is expected given that biomass-attributable PM_2.5_ is a component of all-source PM_2.5_. However, these variables were not included simultaneously in any models. The linear functional form for both all-source and biomass-attributable PM_2.5_ yielded lower Bayesian information criteria and Akaike information criteria values compared to the cubic spline function with 3 df, indicating a better fit. The corresponding exposure–response curves are presented in Figures S6–S8; http://links.lww.com/EE/A335.

Most effect estimates for biomass-attributable and all-source PM_2.5_ exposure and the risk of preterm birth and stillbirth were close to 1.00, with all 95% CI including 1.00 (Table 3, unadjusted OR are shown in Table S13; http://links.lww.com/EE/A335). While there were small variations in point estimates across exposure windows—such as slightly lower estimates for first-trimester all-source PM_2.5_ exposure in preterm births (OR = 0.985, 95% CI = 0.965, 1.009) and slightly higher estimates for the entire pregnancy and third-trimester all-source PM_2.5_ exposure in stillbirths (OR = 1.020, 95% CI = 0.976, 1.066 and OR = 1.030, 95% CI = 0.992, 1.070 respectively)—CI consistently included 1.00, suggesting no consistent evidence of associations. Sensitivity analyses yielded similar null results (Table S14–S16; http://links.lww.com/EE/A335). In the analyses excluding births after October 2019, point estimates for stillbirth were attenuated compared to the main analysis, particularly for all-source PM_2.5_ exposure, however CI in all analyses included the null and were wide (Table S15; http://links.lww.com/EE/A335).

**Table 3.** Preterm birth and stillbirth odds ratios for exposure to biomass-attributable PM_2.5_ and all-source PM_2.5_ across different pregnancy exposure windows

	Biomass-attributable PM_2.5_		All-source PM_2.5_	
Model	Adjusted OR	IQR (μg/m³)	Adjusted OR	IQR (μg/m³)
Preterm birth—entire pregnancy	1.002 (0.997, 1.007)	0.37	1.001 (0.987, 1.015)	1.18
Preterm birth—trimester 1	1.001 (0.992, 1.010)	0.32	0.985 (0.965, 1.009)	1.30
Preterm birth—trimester 2	1.001 (0.998, 1.004)	0.38	1.001 (0.992, 1.009)	1.38
Preterm birth—trimester 3	1.000 (0.998, 1.002)	0.34	0.999 (0.990, 1.008)	1.72
Stillbirth—entire pregnancy	1.002 (0.985, 1.019)	0.37	1.020 (0.976, 1.066)	1.20
Stillbirth—trimester 1	0.992 (0.963, 1.022)	0.31	1.006 (0.943, 1.072)	1.29
Stillbirth—trimester 2	0.998 (0.990, 1.005)	0.33	0.998 (0.971, 1.026)	1.45
Stillbirth—trimester 3	1.006 (0.999, 1.014)	0.31	1.030 (0.992, 1.070)	1.76

OR (with 95% confidence intervals in brackets) were estimated using conditional logistic regression models, adjusted for long-term-trend, month of conception, temperature, relative humidity, and statistical area 2 level socioeconomic quintiles. Controls were matched randomly to cases using a risk-set sampling approach with replacement. Gestational age of controls was truncated to the same age as their matched case. OR are expressed per interquartile range (IQR) increase in exposure. Units are micrograms per cubic meter (μg/m³).

## Discussion

This study assessed the association between ambient biomass-attributable PM_2.5_ and all-source PM_2.5_ exposure and adverse birth outcomes in Sydney, Australia, from 2010 to 2020, considering average exposure levels across the entire pregnancy and individual trimesters to reflect the typical average levels that pregnant women experience. While small variations in point estimates were observed across exposure windows, our findings showed no consistent evidence of associations between either biomass-attributable or all-source PM_2.5_ exposures and preterm births or stillbirths, with CI consistently including the null. This lack of clear associations persisted across different exposure windows and in sensitivity analyses.

Our study has several strengths. It is the first in Australia to focus specifically on biomass-attributable PM_2.5_ and birth outcomes, a critical issue given the increasing frequency of landscape fires and high prevalence of residential wood burning in Australia. Second, it used high-resolution daily air quality data from the Centre for Safe Air’s CARDAT platform. By contrasting biomass-attributable PM_2.5_ with all-source PM_2.5_, we were able to explore the potential differential effects between these two exposure types. Additionally, our study included exposure during an unprecedented wildfire period, which, although not specifically assessed, contributed to the overall extreme longer-term exposure events considered in the analysis. Finally, the application of risk-set sampling and conditional logistic regression helped reduce potential bias by controlling for time-varying exposures. This design was particularly appropriate for examining pregnancy-averaged exposures as it enabled the matching of controls to cases at the same gestational age, ensuring comparable exposure windows while providing interpretable effect estimates for these relatively rare outcomes, as opposed to deriving these effect estimates through Cox Proportional Hazard models, where violation of the proportional hazard model may pose a challenge.

However, several limitations of this analysis should be noted. First, the population-level observational study design is inherently susceptible to residual confounding and exposure misclassification. While residual confounding can obscure the true relationship between PM_2.5_ exposure and adverse birth outcomes in any direction, (nondifferential) exposure misclassification may lead to attenuation of associations. This could occur through several mechanisms: our methodological simplification of assigning exposure based on residential address at delivery, which cannot capture the complex spatiotemporal patterns of individual mobility and activity; and our approach of identifying biomass-attributable PM_2.5_ using days exceeding the 95th percentile of all-source PM_2.5_, which might misclassify some high-pollution days from nonbiomass sources as biomass-attributable, while missing actual biomass pollution events that did not exceed our chosen threshold. Second, survival bias may have underestimated the adverse effect of PM_2.5_ exposure, as our analysis excluded fetal deaths before 20 weeks of gestation, potentially missing early pregnancy losses due to PM_2.5_ exposure.^39^ Third, our study period excluded pregnancies exposed to the unprecedented 2019–2020 wildfires during early gestation, possibly missing effects that could manifest only later or after consistent exposure throughout all three trimesters. Fourth, while our use of trimester averages effectively captured typical exposure patterns throughout pregnancy as intended, different exposure assessment approaches could offer additional insights. Finally, our study design choices, including the use of SA2-level exposure assessment, were guided by our aim to understand population-level associations between typical biomass-attributable PM_2.5_ exposure patterns and birth outcomes. While finer spatial resolution exposure data might be valuable for different research questions, our current approach appropriately matches the resolution of available health data and captures exposure variations relevant to population-level effects.

Our findings are consistent with prior research conducted in Sydney, which generally shows a lack of significant associations between PM_2.5_ and preterm birth or stillbirth. For instance, a study assessing preterm risk in Sydney from 1998 to 2000 found no significant positive associations for various exposure windows (e.g., OR for the first trimester: 0.98, 95% CI = 0.95, 1.01 per 1 μg/m³ increase).^32^ Similarly, a study examining the effects of all-source PM_2.5_ on stillbirths from 1997 to 2012 reported results comparable to ours, with an OR for entire pregnancy exposure of 1.02 (95% CI = 0.97, 1.06) per 2 μg/m³ increase.^33^ Our study builds on and extends the findings of Brew et al.,^25^ who compared pregnancies exposed during the 2019–2020 wildfires in southeast Sydney with matching periods in the two years prior. They found no clear association with preterm births (OR = 0.95; 95% CI = 0.88, 1.02) or stillbirths (OR = 1.11; 95% CI = 0.84, 1.46).

Nonetheless, there are studies that reported adverse effects of wildfire smoke exposure on birth outcomes. For instance, a California study reported that each additional day of wildfire smoke exposure during pregnancy was associated with a 0.49% (95% CI = 0.41%, 0.59%) increase in the risk of preterm birth. Interestingly, the study found no association with preterm birth for low-intensity smoke days, but only above medium-intensity smoke days.^20^ Zhang, et al^23^ reported a hazard ratio of 1.07 (95% CI = 1.06, 1.08) for preterm births per IQR (0.85 μg/m³) increase in wildfire smoke-specific PM_2.5_ exposure in NSW from 2016 to 2019, employing chemical transport modeling combined with machine-learning and satellite data. Another study from NSW examined short-term wildfire-specific PM_2.5_ exposure and adverse perinatal outcomes from 2009 to 2015 during wildfire seasons (August—December), estimating daily wildfire-specific PM_2.5_ using satellite observations, and a machine-learning and Stochastic Time-Inverted Lagrangian Transport model. While unadjusted hazard ratios showed no clear associations for exposure during the last gestational week, they found in adjusted models hazard ratios of 1.17 (95% CI = 1.04, 1.32) for preterm birth and 1.40 (95% CI = 1.11, 1.78) for stillbirth per 10 μg/m³ increase in wildfire-specific PM_2.5_.^24^ Finally, a case-crossover study across different regions in Brazil found positive associations between wildfire exposure and preterm birth,^21^ though their region-specific exposure classification based on the 90th percentile of both wildfire events and PM_2.5_ concentrations may have led to exposure misclassification. This was particularly evident in the Southeast region of Brazil, which reported the highest OR (1.41; 95% CI = 1.31, 1.51) despite few wildfire events, suggesting possible confounding from other more prevalent sources in that region, such as urban and industrial pollution.^53^

These contrasting findings compared with our study are likely due to several key methodological differences. First, air pollution modeling approaches, confounder adjustments, and statistical models employed differed across all the studies. Second, exposure assessment approaches varied substantially between studies. While our study used trimester and pregnancy averages to understand typical exposure patterns, other studies have focused on the frequency of high-pollution days or short-term and critical exposure windows.^20,24^ Our approach of averaging exposures over trimesters, while suited to examining chronic exposure patterns, may have missed critical exposure windows and diluted the effects of intense but brief pollution episodes. This is particularly relevant in Sydney, where biomass-attributable events, although producing high daily PM_2.5_ concentrations, may not have reached critical levels to detect population-level effects when averaged over longer periods. While there is emerging evidence that no level of air pollution is entirely safe for many health outcomes,^54^ the exposure–response relationship for adverse birth outcomes requires further characterization,^55^ particularly regarding potential thresholds,^56,57^ the relative importance of acute versus chronic exposures and which exposure metrics best capture biologically relevant patterns. Studies incorporating multiple approaches for exposure assessment could help identify the most relevant exposure patterns.

While our study did not find evidence of an association between biomass-attributable PM_2.5_ or all-source PM_2.5_ exposure and preterm births or stillbirths, the hypothesized biological mechanisms linking PM_2.5_ exposure to adverse birth outcomes—such as inflammation, oxidative stress, and placental dysfunction—remain plausible.^3,18,58^ Furthermore, it is important to note that pregnant women and their children may still be impacted by these risk factors through other adverse health effects.^1,2,44,59–68^ Our findings are specific to metropolitan Sydney from 2010 to 2020 and may not be generalizable to other regions with different environmental, demographic, and socioeconomic contexts.

## Conclusions

While biomass-attributable PM_2.5_ has been hypothesized to be more harmful due to its chemical composition, our findings from Sydney suggest no clear evidence of increased risk of preterm birth or stillbirth, when examining average pregnancy or trimester-specific exposures to biomass-attributable PM_2.5_. However, our exposure metric may not capture the potential effects of critical windows, short-term high exposure events, or threshold exceedances. The null findings of our study add to the limited and mixed evidence on this public health concern. The mixed evidence underscores the complexity of establishing clear exposure-response relationships in environmental epidemiology, making it challenging to interpret and provide recommendations. As climate change will increase the frequency and intensity of landscape fires, continued research using various exposure metrics and examining different temporal patterns of exposure is essential to better understand the potential health impacts of extreme pollution events.

## CONFLICTS OF INTEREST STATEMENTS

The authors declare that they have no conflicts of interest with regard to the content of this report.

## ACKNOWLEDGMENTS

The authors thank the NSW Ministry of Health for providing access to the Perinatal Data Collection and the Centre for Safe Air for providing air pollution and temperature data from Centre for Air Pollution, Energy, and Health Research Data Platform (CARDAT) funded by the NHMRC Centre for Safe Air (CAR; https://www.car-cre.org.au/; https://cardat.github.io) which also received funding from the Australian Research Data Commons (ARDC) for the Integrated National Air Pollution and Health Data project https://doi.org/10.47486/PS022. This research includes computations using the computational cluster Katana supported by Research Technology Services at University of New South Wales Sydney. We thank Tanya Nippita from the University of Sydney Northern Clinical School, Women and Babies Research, St Leonards, NSW, Australia and Northern Sydney Local Health District, Department of Obstetrics and Gynecology, Royal North Shore Hospital, Sydney, NSW, Australia, and Deborah Randall from the University of Sydney Northern Clinical School, Women and Babies Research, St Leonards, NSW, Australia for their input on obstetrics related questions at the early stages of this study.

## Supplementary Material



## References

[1] Zang ST Wu QJ Li XY. Long-term PM2.5 exposure and various health outcomes: An umbrella review of systematic reviews and meta-analyses of observational studies. Sci Total Environ. 2022;812:152381. 34914980 10.1016/j.scitotenv.2021.152381

[2] Orellano P Reynoso J Quaranta N Bardach A Ciapponi A. Short-term exposure to particulate matter (PM10 and PM2.5), nitrogen dioxide (NO2), and ozone (O3) and all-cause and cause-specific mortality: systematic review and meta-analysis. Environ Int. 2020;142:105876. 32590284 10.1016/j.envint.2020.105876

[3] Song S Gao Z Zhang X. Ambient fine particulate matter and pregnancy outcomes: An umbrella review. Environ Res. 2023;235:116652. 37451569 10.1016/j.envres.2023.116652

[4] Zhang H Zhang X Wang Q. Ambient air pollution and stillbirth: an updated systematic review and meta-analysis of epidemiological studies. Environ Pollut. 2021;278:116752. 33689950 10.1016/j.envpol.2021.116752

[5] Black C Tesfaigzi Y Bassein JA Miller LA. Wildfire smoke exposure and human health: significant gaps in research for a growing public health issue. Environ Toxicol Pharmacol. 2017;55:186–195. 28892756 10.1016/j.etap.2017.08.022 PMC5628149

[6] Reid CE Brauer M Johnston FH Jerrett M Balmes JR Elliott CT. Critical review of health impacts of wildfire smoke exposure. Environ Health Perspect. 2016;124:1334–1343. 27082891 10.1289/ehp.1409277 PMC5010409

[7] Lelieveld J Evans JS Fnais M Giannadaki D Pozzer A. The contribution of outdoor air pollution sources to premature mortality on a global scale. Nature. 2015;525:367–371. 26381985 10.1038/nature15371

[8] Borchers-Arriagada N Vander Hoorn S Cope M. The mortality burden attributable to wood heater smoke particulate matter (PM2.5) in Australia. Sci Total Environ. 2024;921:171069. 38395157 10.1016/j.scitotenv.2024.171069

[9] Jiang K Xing R Luo Z. Pollutant emissions from biomass burning: a review on emission characteristics, environmental impacts, and research perspectives. Particuology. 2024;85:296–309.

[10] Wegesser TC Pinkerton KE Last JA. California wildfires of 2008: coarse and fine particulate matter toxicity. Environ Health Perspect. 2009;117:893–897. 19590679 10.1289/ehp.0800166 PMC2702402

[11] Johnston FH Williamson G Borchers-Arriagada N Henderson SB Bowman DMJS. Climate change, landscape fires, and human health: a global perspective. Annu Rev Public Health. 2024;45:295. 38166500 10.1146/annurev-publhealth-060222-034131

[12] Aguilera R Corringham T Gershunov A Benmarhnia T. Wildfire smoke impacts respiratory health more than fine particles from other sources: observational evidence from Southern California. Nat Commun. 2021;12:1493. 33674571 10.1038/s41467-021-21708-0 PMC7935892

[13] Scott AF Reilly CA. Wood and biomass smoke: addressing human health risks and exposures. Chem Res Toxicol. 2019;32:219–221. 30721037 10.1021/acs.chemrestox.8b00318

[14] Karanasiou A Alastuey A Amato F. Short-term health effects from outdoor exposure to biomass burning emissions: a review. Sci Total Environ. 2021;781:146739. 33798874 10.1016/j.scitotenv.2021.146739

[15] Sigsgaard T Forsberg B Annesi-Maesano I. Health impacts of anthropogenic biomass burning in the developed world. Eur Respir J. 2015;46:1577–1588. 26405285 10.1183/13993003.01865-2014

[16] Johnston HJ Mueller W Steinle S. How harmful is particulate matter emitted from biomass burning? A Thailand perspective. Curr Pollut Rep. 2019;5:353–377.

[17] Amjad S Chojecki D Osornio-Vargas A Ospina MB. Wildfire exposure during pregnancy and the risk of adverse birth outcomes: a systematic review. Environ Int. 2021;156:106644. 34030071 10.1016/j.envint.2021.106644

[18] Foo D Stewart R Heo S. Wildfire smoke exposure during pregnancy and perinatal, obstetric, and early childhood health outcomes: a systematic review and meta-analysis. Environ Res. 2024;241:117527. 37931734 10.1016/j.envres.2023.117527

[19] Evans J Bansal A Schoenaker DAJM Cherbuin N Peek MJ Davis DL. Birth outcomes, health, and health care needs of childbearing women following wildfire disasters: an integrative, state-of-the-science review. Environ Health Perspect. 2022;130:86001. 35980335 10.1289/EHP10544 PMC9387511

[20] Heft-Neal S Driscoll A Yang W Shaw G Burke M. Associations between wildfire smoke exposure during pregnancy and risk of preterm birth in California. Environ Res. 2022;203:111872. 34403668 10.1016/j.envres.2021.111872

[21] Requia WJ Papatheodorou S Koutrakis P Mukherjee R Roig HL. Increased preterm birth following maternal wildfire smoke exposure in Brazil. Int J Hyg Environ Health. 2022;240:113901. 34891058 10.1016/j.ijheh.2021.113901

[22] Jones BA McDermott S. Infant health outcomes in mega-fire affected communities. Appl Econ Letters. 2022;29:1325–1335.

[23] Zhang Y Ye T Yu P. Preterm birth and term low birth weight associated with wildfire-specific PM2.5: a cohort study in New South Wales, Australia during 2016–2019. Environ Int. 2023;174:107879. 36958111 10.1016/j.envint.2023.107879

[24] Nyadanu SD Foo D Pereira G Mickley LJ Feng X Bell ML. Short-term effects of wildfire-specific fine particulate matter and its carbonaceous components on perinatal outcomes: a multicentre cohort study in New South Wales, Australia. Environ Int. 2024;191:109007. 39278048 10.1016/j.envint.2024.109007

[25] Brew BK Donnolley N Henry A Dahlen H Jalaludin B Chambers GM. Double jeopardy-pregnancy and birth during a catastrophic bushfire event followed by a pandemic lockdown, a natural experiment. Environ Res. 2022;214:113752. 35777439 10.1016/j.envres.2022.113752

[26] Emmerson KM Keywood MD. Air quality: Outlook and impacts. Australia State of the environment 2021. Canberra: Australian Government Department of Agriculture, Water and the Environment, 2021.

[27] NSW Government. Air quality study for the NSW Greater Metropolitan Region. Sydney: State of NSW and Department of Planning, Industry and Environment, 2020.

[28] IQAir. 2019 World Air Quality Report. In: IQAir, ed. Vol. 8, 2020.

[29] Johnston FH. Burning to reduce fuels: the benefits and risks of a public health protection strategy. Med J Aust. 2020;213:246–248.e1. 32864743 10.5694/mja2.50751

[30] Singh T Jalaludin B Hajat S. Acute air pollution and temperature exposure as independent and joint triggers of spontaneous preterm birth in New South Wales, Australia: a time-to-event analysis. Front Public Health. 2023;11:1220797. 38098836 10.3389/fpubh.2023.1220797 PMC10720724

[31] Chen G Guo Y Abramson MJ Williams G Li S. Exposure to low concentrations of air pollutants and adverse birth outcomes in Brisbane, Australia, 2003-2013. Sci Total Environ. 2018;622-623:721–726. 29223898 10.1016/j.scitotenv.2017.12.050

[32] Jalaludin B Mannes T Morgan G Lincoln D Sheppeard V Corbett S. Impact of ambient air pollution on gestational age is modified by season in Sydney, Australia. Environ Health. 2007;6:16. 17553174 10.1186/1476-069X-6-16 PMC1894960

[33] Jalaludin B Salimi F Sadeghi M Collie L Morgan G. Ambient air pollution and stillbirths risk in Sydney, Australia. Toxics. 2021;9:209. 34564360 10.3390/toxics9090209 PMC8473280

[34] Australian Bureau of Statistics. Greater Sydney. https://www.abs.gov.au/census/find-census-data/quickstats/2021/1GSYD. Accessed 24 November 2024.

[35] Filkov AI Ngo T Matthews S Telfer S Penman TD. Impact of Australia’s catastrophic 2019/20 bushfire season on communities and environment. Retrospective analysis and current trends. J Saf Sci Resil. 2020;1:44–56.

[36] Centre for Epidemiology and Evidence. New South Wales mothers and babies 2020. In: Health NMo, ed. New South Wales Mothers and Babies. Sydney: NSW Ministry of Health, 2021.

[37] Australian Bureau of Statistics. Socio-economic indexes for areas (SEIFA), Australia. In: Australia Co, ed. Canberra, 2021.

[38] Strand LB Barnett AG Tong S. Methodological challenges when estimating the effects of season and seasonal exposures on birth outcomes. BMC Med Res Methodol. 2011;11:49–49. 21501523 10.1186/1471-2288-11-49 PMC3102035

[39] Neophytou AM Kioumourtzoglou M-A Goin DE Darwin KC Casey JA. Educational note: addressing special cases of bias that frequently occur in perinatal epidemiology. Int J Epidemiol. 2020;50:337–345. 10.1093/ije/dyaa252 PMC8453403 33367719

[40] Iwagami M Shinozaki T. Introduction to matching in case-control and cohort studies. Ann Clin Epidemiol. 2022;4:33–40. 38504854 10.37737/ace.22005 PMC10760465

[41] Wilhelm M Ghosh JK Su J Cockburn M Jerrett M Ritz B. Traffic-related air toxics and preterm birth: a population-based case-control study in Los Angeles county, California. Environ Health. 2011;10:89. 21981989 10.1186/1476-069X-10-89 PMC3204282

[42] Hanigan I Yuen C Gopi K Borchers-Arriagada N van Buskirk J Morgan G. Bushfire specific PM2.5 output from v1.3 based on satellite and other land use and other predictors for Australia 2001-2020 produced for the CAR Bushfire Smoke Exposures project. In: CAR, ed. CAR. Centre for Air pollution, energy and health Research, 2023.

[43] Borchers-Arriagada N Morgan GG Van Buskirk J. Daily PM2.5 and seasonal-trend decomposition to identify extreme air pollution events from 2001 to 2020 for Continental Australia using a random forest model. Atmosphere. 2024;15:1341.

[44] Jegasothy E Hanigan IC Van Buskirk J. Acute health effects of bushfire smoke on mortality in Sydney, Australia. Environ Int. 2023;171:107684. 36577296 10.1016/j.envint.2022.107684

[45] Australian Bureau of Meteorology. Australian Gridded Climate Data (AGCD)/ AWAP; v1.0.0 Snapshot (1900-01-01 to 2020-06-30). In: Meteorology ABo, ed. 1.0.0 ed, 2020.

[46] Laurent O Hu J Li L. Statewide nested case–control study of preterm birth and air pollution by source and composition: California, 2001–2008. Environ Health Perspect. 2016;124:1479–1486. 26895492 10.1289/ehp.1510133 PMC5010414

[47] Australian Institute of Health and Welfare. Stillbirths and neonatal deaths in Australia 2017 and 2018. Perinatal statistics series no 38. Canberra: AIHW, 2021.

[48] Australian Institute of Health and Welfare. Australia’s mothers and babies. https://www.aihw.gov.au/reports/mothers-babies/australias-mothers-babies/contents/baby-outcomes/gestational-age Accessed 13 May 2024.

[49] Australian Institute of Health and Welfare. National Perinatal Mortality Data Collection. https://www.aihw.gov.au/about-our-data/our-data-collections/national-perinatal-mortality-data-collection-npmdc#:~:text=The%20National%20Perinatal%20Mortality%20Data,gestation%20or%20400%20grams%20birthweight. Accessed 13 May 2024.

[50] Greenland S Pearl J Robins JM. Causal diagrams for epidemiologic research. Epidemiology. 1999;10:37–48. 9888278

[51] Therneau TM. A package for survival analysis in R. 2020.

[52] Bates M Venables B Team MRC. Package “splines. R Version. 2011;2.

[53] Andrade MF Ynoue RY Freitas ED. Air quality forecasting system for Southeastern Brazil. Front Environ Sci. 2015;3:9.

[54] Hoffmann B Boogaard H de Nazelle A. WHO Air Quality Guidelines 2021–aiming for healthier air for all: a joint statement by medical, public health, scientific societies and patient representative organisations. Int J Public Health. 2021;66:1604465. 34630006 10.3389/ijph.2021.1604465 PMC8494774

[55] Balakrishnan K Steenland K Clasen T; HAPIN Investigators. Exposure–response relationships for personal exposure to fine particulate matter (PM_2.5_), carbon monoxide, and black carbon and birthweight: an observational analysis of the multicountry Household Air Pollution Intervention Network (HAPIN) trial. Lancet Planetary Health. 2023;7:e387–e396. 37164515 10.1016/S2542-5196(23)00052-9 PMC10186177

[56] Ming X He Z Li Y. The short-term effects of air pollution exposure on preterm births in Chongqing, China: 2015–2020. Environ Sci Pollut Res Int. 2023;30:51679–51691. 36810823 10.1007/s11356-023-25624-2 PMC10119072

[57] Xiao X Liu R Yu Y. Evidence of interactive effects of late-pregnancy exposure to air pollution and extreme temperature on preterm birth in China: a nationwide study. Environ Res Lett. 2023;18:094017.

[58] Basilio E Chen R Fernandez AC Padula AM Robinson JF Gaw SL. Wildfire smoke exposure during pregnancy: a review of potential mechanisms of placental toxicity, impact on obstetric outcomes, and strategies to reduce exposure. Int J Environ Res Public Health. 2022;19:13727. 36360613 10.3390/ijerph192113727 PMC9657128

[59] Thangavel P Park D Lee Y-C. Recent insights into particulate matter (PM2.5)-mediated toxicity in humans: an overview. Int J Environ Res Public Health. 2022;19:7511. 35742761 10.3390/ijerph19127511 PMC9223652

[60] Chen J Hoek G. Long-term exposure to PM and all-cause and cause-specific mortality: a systematic review and meta-analysis. Environ Int. 2020;143:105974. 32703584 10.1016/j.envint.2020.105974

[61] Liu C Chen R Sera F. Ambient particulate air pollution and daily mortality in 652 cities. N Engl J Med. 2019;381:705–715. 31433918 10.1056/NEJMoa1817364 PMC7891185

[62] Martin KL Hanigan IC Morgan GG Henderson SB Johnston FH. Air pollution from bushfires and their association with hospital admissions in Sydney, Newcastle and Wollongong, Australia 1994–2007. Aust N Z J Public Health. 2013;37:238–243. 23731106 10.1111/1753-6405.12065

[63] Jalaludin B Khalaj B Sheppeard V Morgan G. Air pollution and ED visits for asthma in Australian children: a case-crossover analysis. Int Arch Occup Environ Health. 2008;81:967–974. 18094989 10.1007/s00420-007-0290-0

[64] Shao J Zosky GR Hall GL. Early life exposure to coal mine fire smoke emissions and altered lung function in young children. Respirology. 2020;25:198–205. 31231911 10.1111/resp.13617

[65] Melody SM Ford JB Wills K Venn A Johnston FH. Maternal exposure to fine particulate matter from a large coal mine fire is associated with gestational diabetes mellitus: a prospective cohort study. Environ Res. 2020;183:108956. 31831154 10.1016/j.envres.2019.108956

[66] Guo Y Gao CX Dennekamp M. The association of coal mine fire smoke with hospital emergency presentations and admissions: time series analysis of Hazelwood Health Study. Chemosphere. 2020;253:126667. 32278916 10.1016/j.chemosphere.2020.126667

[67] Hanigan IC Rolfe MI Knibbs LD. All-cause mortality and long-term exposure to low level air pollution in the “45 and up study” cohort, Sydney, Australia, 2006–2015. Environ Int. 2019;126:762–770. 30878871 10.1016/j.envint.2019.02.044

[68] Salimi F Morgan G Rolfe M. Long-term exposure to low concentrations of air pollutants and hospitalisation for respiratory diseases: a prospective cohort study in Australia. Environ Int. 2018;121:415–420. 30261462 10.1016/j.envint.2018.08.050

