# Supplementary material for: Quantifying the association between pregnancy exposure to biomass-attributable PM2.5 and the risk of preterm birth and stillbirth: A case-control study in Sydney, Australia for 2010–2020
Source: Environ Epidemiol. 2025 Mar 26;9(2):e381. doi: 10.1097/EE9.0000000000000381 (PMC11949295; doi:10.1097/EE9.0000000000000381)
Supplement: Supplementary file 1 [file ee9-9-e381-s001.pdf]

Supplementary Material: Quantifying the Association Between Pregnancy Exposure to Biomass-Attributable $PM_{2.5}$ and the Risk of Preterm Birth and Stillbirth: A Case-Control Study in Sydney, Australia for 2010–2020

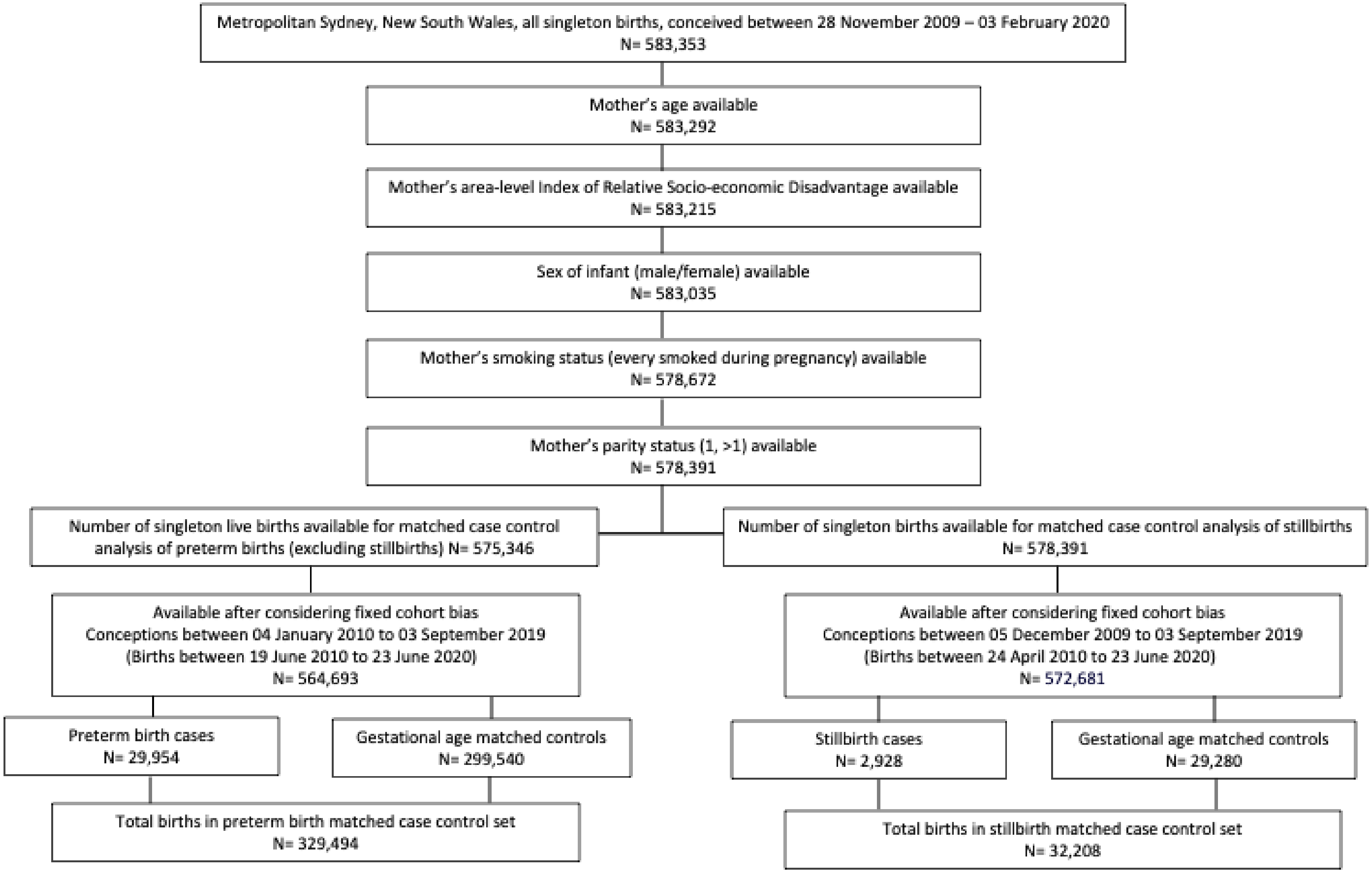

Figure S1: Flowchart of inclusion and exclusion criteria for study population

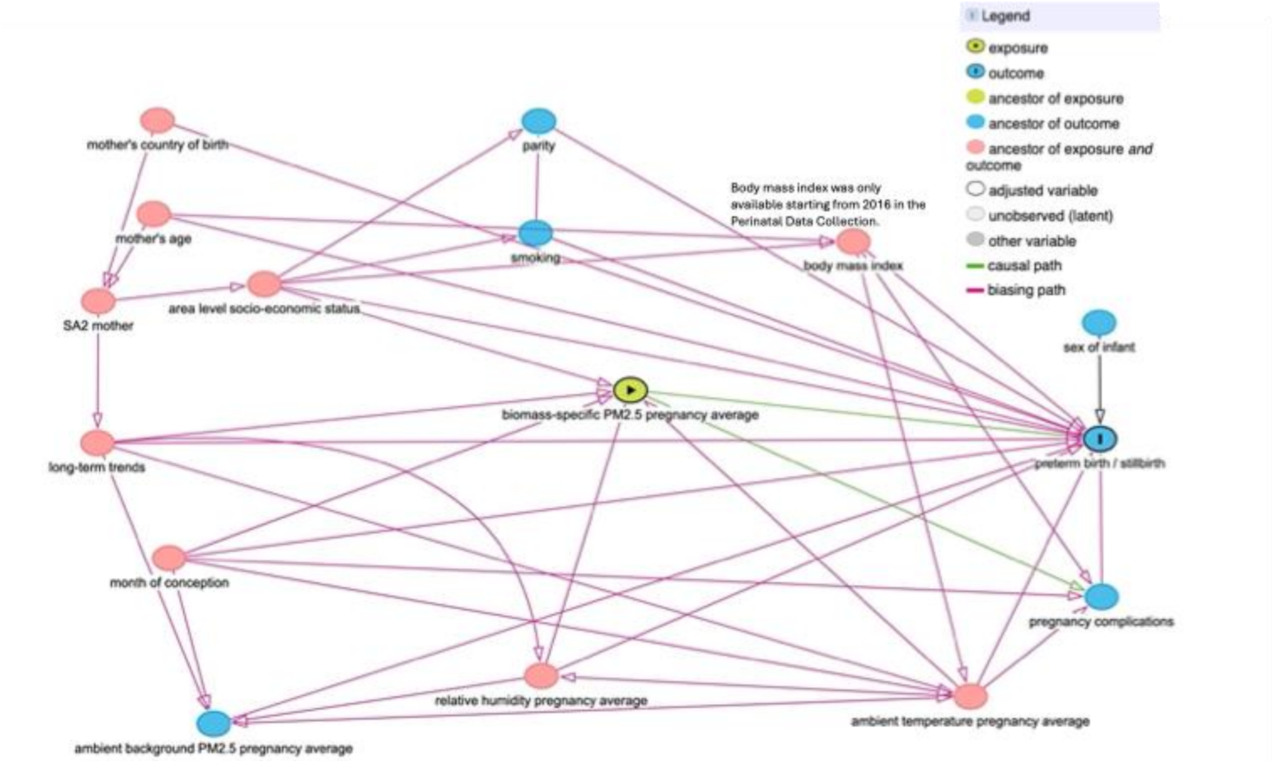

Minimal sufficient adjustment sets for estimating the total effect of exposure to ambient biomass-specific $PM_{2.5}$ during pregnancy on preterm birth / stillbirth: **ambient temperature pregnancy average, area level socio-economic status, long-term trends, relative humidity pregnancy average, season of conception**

Figure S2: Directed acyclic graph (DAG) for minimum sufficient sets

Table S1: Preterm birth matched case control set by birth outcome and gestational age (N and percentages)

GESTAGE	Term birth (%)	Preterm birth (%)	Term/control births (N)	Preterm birth (N)	Cumulative term birth (%)	Cumulative preterm birth (%)	Cumulative term birth (N)	Cumulative preterm birth (N)
20	0	0.37	0	112	0	0.37	0	112
21	0	0.44	0	132	0	0.81	0	244
22	0	0.56	0	169	0	1.37	0	413
23	0	0.52	2	155	0	1.89	2	568
24	0	0.71	3	212	0	2.6	5	780
25	0	0.91	3	274	0	3.51	8	1,054
26	0	1.04	5	311	0	4.55	13	1,365
27	0	1.18	6	352	0	5.73	19	1,717
28	0.01	1.5	15	450	0.01	7.23	34	2,167
29	0.01	1.6	15	478	0.02	8.83	49	2,645
30	0.01	2.27	35	681	0.03	11.1	84	3,326
31	0.01	2.68	40	804	0.04	13.78	124	4,13
32	0.03	4.27	87	1,278	0.07	18.05	211	5,408
33	0.06	6.53	189	1,956	0.13	24.58	400	7,364
34	0.15	11.49	446	3,441	0.28	36.07	846	10,805
35	0.4	20.15	1,191	6,035	0.68	56.22	2,037	16,84
36	1.33	43.78	3,969	13,114	2.01	100	6,006	29,954
37	7.07	0	21,19	0	9.08	100	27,196	29,954
38	20.37	0	61,009	0	29.45	100	88,205	29,954
39	33.5	0	100,336	0	62.95	100	188,541	29,954
40	25.07	0	75,08	0	88.02	100	263,621	29,954
41	11.54	0	34,569	0	99.56	100	298,19	29,954
42	0.43	0	1,288	0	99.99	100	299,478	29,954
43	0.02	0	62	0	100.01	100	299,54	29,954

Table S2: Stillbirth matched case control set by birth outcome and gestational age (N and percentages)

GESTAGE	Live/control births (%)	Stillbirths (%)	Live/control births (N)	Stillbirths (N)	Cumulative live birth (%)	Cumulative stillbirth %	Cumulative live birth N	Cumulative stillbirth N
20	0	12.57	1	368	0	12.57	1	368
21	0.02	12.3	5	360	0.02	24.87	6	728
22	0.04	9.77	12	286	0.06	34.64	18	1,014
23	0.02	6.83	7	200	0.08	41.47	25	1,214
24	0.01	4.64	4	136	0.09	46.11	29	1,35
25	0.04	4.64	12	136	0.13	50.75	41	1,486
26	0.03	3.45	10	101	0.16	54.2	51	1,587
27	0.05	3.24	14	95	0.21	57.44	65	1,682
28	0.04	2.66	13	78	0.25	60.1	78	1,76
29	0.07	2.49	20	73	0.32	62.59	98	1,833
30	0.1	2.19	28	64	0.42	64.78	126	1,897
31	0.1	2.22	28	65	0.52	67	154	1,962
32	0.15	2.39	45	70	0.67	69.39	199	2,032
33	0.26	2.32	75	68	0.93	71.71	274	2,1
34	0.45	2.49	132	73	1.38	74.2	406	2,173
35	0.83	2.36	242	69	2.21	76.56	648	2,242
36	1.92	4.51	562	132	4.13	81.07	1,21	2,374
37	6.01	4.17	1,759	122	10.14	85.24	2,969	2,496
38	17.48	4.51	5,119	132	27.62	89.75	8,088	2,628
39	31.19	4.58	9,133	134	58.81	94.33	17,221	2,762
40	26.26	3.38	7,688	99	85.07	97.71	24,909	2,861
41	14.13	2.02	4,137	59	99.2	99.73	29,046	2,92
42	0.74	0.24	217	7	99.94	99.97	29,263	2,927
43	0.06	0.03	17	1	100	100	29,28	2,928

Table S3: Summary statistics of exposure variables for preterm birth and stillbirth matched sets during different pregnancy windows

Exposure and pregnancy window	Preterm birth set [a]	Minimum	5th	25th	Median	75th	95th	99th	Maximum	Stillbirth set [b]	Minimum	5th	25th	Median	75th	95th	99th	Maximum
All-source $PM_{2.5}$ entire pregnancy	**Control**	5.13	6.16	6.95	7.57	8.13	9.26	14.04	21.08	**Control**	5.04	6.08	6.91	7.51	8.11	9.23	14.00	25.86
	**Preterm birth**	5.29	6.16	6.93	7.57	8.14	9.29	14.13	19.05	**Stillbirth**	5.10	6.12	6.99	7.56	8.17	9.33	14.49	19.14
All-source $PM_{2.5}$ trimester 1	**Control**	4.74	5.85	6.82	7.41	8.11	9.38	10.28	17.31	**Control**	4.76	5.83	6.80	7.39	8.09	9.36	10.25	16.35
	**Preterm birth**	4.81	5.84	6.80	7.40	8.11	9.41	10.33	15.70	**Stillbirth**	4.94	5.84	6.86	7.42	8.15	9.42	10.31	15.89
All-source $PM_{2.5}$ trimester 2	**Control**	4.69	5.86	6.84	7.47	8.22	9.85	20.67	37.93	**Control**	4.45	5.78	6.76	7.44	8.22	9.94	21.50	59.30
	**Preterm birth**	4.80	5.86	6.83	7.46	8.24	9.89	20.63	34.16	**Stillbirth**	4.84	5.83	6.84	7.51	8.26	10.29	22.52	29.99
All-source $PM_{2.5}$ trimester 3	**Control**	2.67	5.60	6.62	7.44	8.34	10.53	23.57	95.52	**Control**	2.88	5.48	6.60	7.43	8.36	10.66	22.94	62.25
	**Preterm birth**	3.01	5.61	6.63	7.44	8.35	10.52	23.81	61.41	**Stillbirth**	3.44	5.52	6.67	7.44	8.40	11.09	26.08	48.84
Non-biomass-attributable $PM_{2.5}$ entire pregnancy	**Control**	5.13	6.11	6.82	7.35	7.73	8.23	8.54	9.25	**Control**	5.04	6.04	6.80	7.32	7.72	8.26	8.65	9.46
	**Preterm birth**	5.29	6.10	6.81	7.35	7.74	8.24	8.54	9.17	**Stillbirth**	5.10	6.08	6.87	7.37	7.77	8.33	8.73	9.38

Exposure and pregnancy window	Preterm birth set [a]	Minimum	5th	25th	Median	75th	95th	99th	Maximum	Stillbirth set [b]	Minimum	5th	25th	Median	75th	95th	99th	Maximum
Non-biomass-attributable $PM_{2.5}$ trimester 1	**Control**	4.74	5.82	6.73	7.25	7.75	8.49	9.00	9.95	**Control**	4.76	5.80	6.72	7.24	7.74	8.49	9.02	9.88
	**Preterm birth**	4.81	5.81	6.72	7.25	7.75	8.51	9.00	9.93	**Stillbirth**	4.94	5.82	6.78	7.27	7.79	8.58	8.97	9.69
Non-biomass-attributable $PM_{2.5}$ trimester 2	**Control**	4.69	5.83	6.76	7.30	7.81	8.66	9.36	11.21	**Control**	4.45	5.76	6.69	7.28	7.84	8.74	9.51	11.86
	**Preterm birth**	4.80	5.82	6.75	7.29	7.82	8.67	9.37	10.67	**Stillbirth**	4.84	5.82	6.76	7.33	7.88	8.87	9.53	11.02
Non-biomass-attributable $PM_{2.5}$ trimester 3	**Control**	2.67	5.58	6.55	7.27	7.96	9.05	10.15	40.06	**Control**	2.88	5.47	6.54	7.26	7.96	9.15	10.66	27.52
	**Preterm birth**	3.01	5.59	6.55	7.27	7.98	9.06	10.17	55.19	**Stillbirth**	3.44	5.50	6.60	7.28	7.99	9.38	10.88	12.40
Biomass -attributable $PM_{2.5}$ entire pregnancy	**Control**	0.00	0.00	0.07	0.19	0.44	1.13	5.91	12.86	**Control**	0.00	0.00	0.05	0.15	0.42	1.01	5.92	17.14
	**Preterm birth**	0.00	0.00	0.07	0.18	0.44	1.13	5.98	10.58	**Stillbirth**	0.00	0.00	0.05	0.16	0.43	1.06	6.36	10.50
Biomass -attributable	**Control**	0.00	0.00	0.00	0.11	0.32	1.14	1.93	9.89	**Control**	0.00	0.00	0.00	0.10	0.31	1.13	1.84	8.54
	**Preterm birth**	0.00	0.00	0.00	0.10	0.32	1.16	1.97	8.43	**Stillbirth**	0.00	0.00	0.00	0.11	0.32	1.17	2.05	8.46

Exposure and pregnancy window	Preterm birth set [a]	Minimum	5th	25th	Median	75th	95th	99th	Maximum	Stillbirth set [b]	Minimum	5th	25th	Median	75th	95th	99th	Maximum
$PM_{2.5}$ trimester 1																		
Biomass - attributable $PM_{2.5}$ trimester 2	**Control**	0.00	0.00	0.00	0.11	0.38	1.45	11.59	27.48	**Control**	0.00	0.00	0.00	0.10	0.33	1.53	12.15	47.44
	**Preterm birth**	0.00	0.00	0.00	0.11	0.37	1.48	11.55	23.75	**Stillbirth**	0.00	0.00	0.00	0.10	0.34	1.62	13.29	19.62
Biomass - attributable $PM_{2.5}$ trimester 3	**Control**	0.00	0.00	0.00	0.00	0.33	1.97	14.12	89.14	**Control**	0.00	0.00	0.00	0.00	0.31	1.89	13.41	55.62
	**Preterm birth**	0.00	0.00	0.00	0.00	0.34	1.91	14.15	53.98	**Stillbirth**	0.00	0.00	0.00	0.00	0.36	2.03	16.19	41.13
Number of biomass events entire pregnancy	**Control**	0.00	0.00	3.00	6.00	12.00	26.00	63.00	85.00	**Control**	0.00	0.00	1.00	4.00	10.00	20.00	57.00	82.00
	**Preterm birth**	0.00	0.00	2.00	6.00	12.00	26.00	64.00	80.00	**Stillbirth**	0.00	0.00	2.00	5.00	10.00	21.00	59.73	80.00
Number of biomass events trimester 1	**Control**	0.00	0.00	0.00	2.00	4.00	12.00	17.00	30.00	**Control**	0.00	0.00	0.00	2.00	4.00	12.00	17.00	28.00
	**Preterm birth**	0.00	0.00	0.00	2.00	4.00	12.00	17.00	26.00	**Stillbirth**	0.00	0.00	0.00	2.00	4.00	11.65	17.00	22.00
	**Control**	0.00	0.00	0.00	2.00	5.00	15.00	48.00	74.00	**Control**	0.00	0.00	0.00	1.00	4.00	12.00	39.00	70.00

Exposure and pregnancy window	Preterm birth set [a]	Minimum	5th	25th	Median	75th	95th	99th	Maximum	Stillbirth set [b]	Minimum	5th	25th	Median	75th	95th	99th	Maximum
Number of biomass events trimester 2	**Preterm birth**	0.00	0.00	0.00	2.00	5.00	15.00	47.00	72.00	**Stillbirth**	0.00	0.00	0.00	1.00	4.00	13.00	40.73	60.00
Number of biomass events trimester 3	**Control**	0.00	0.00	0.00	0.00	2.00	9.00	28.00	49.00	**Control**	0.00	0.00	0.00	0.00	2.00	10.00	31.00	59.00
	**Preterm birth**	0.00	0.00	0.00	0.00	2.00	9.00	29.00	49.00	**Stillbirth**	0.00	0.00	0.00	0.00	3.00	10.75	34.55	56.00
Relative humidity entire pregnancy	**Control**	53.92	60.53	63.51	66.65	69.03	71.34	73.29	77.24	**Control**	53.67	59.22	63.45	67.06	69.39	72.23	74.17	77.78
	**Preterm birth**	54.80	60.40	63.47	66.62	69.01	71.24	73.21	76.28	**Stillbirth**	53.81	59.00	63.13	66.62	69.16	72.02	73.82	76.03
Relative humidity trimester 1	**Control**	50.64	56.52	63.47	67.37	70.34	73.17	75.25	79.55	**Control**	50.84	56.59	63.54	67.45	70.38	73.27	75.45	79.08
	**Preterm birth**	50.70	56.42	63.31	67.31	70.28	73.16	75.22	78.87	**Stillbirth**	50.94	56.55	63.00	67.17	70.18	73.02	74.84	77.75
Relative humidity trimester 2	**Control**	46.25	56.02	62.99	67.07	70.21	73.20	75.28	81.22	**Control**	45.57	55.30	63.02	67.24	70.53	73.87	76.21	81.40
	**Preterm birth**	47.38	55.92	62.95	67.04	70.17	73.16	75.20	78.84	**Stillbirth**	46.89	54.97	62.58	66.83	70.32	73.58	75.60	79.04
	**Control**	21.47	53.60	62.47	67.16	70.78	74.87	78.11	105.69	**Control**	25.90	53.58	62.25	67.05	70.78	75.58	81.67	103.18

Exposure and pregnancy window	Preterm birth set [a]	Minimum	5th	25th	Median	75th	95th	99th	Maximum	Stillbirth set [b]	Minimum	5th	25th	Median	75th	95th	99th	Maximum
Relative humidity trimester 3	**Preterm birth**	23.72	53.67	62.45	67.16	70.76	74.81	77.89	100.31	**Stillbirth**	34.70	53.38	61.88	66.76	70.54	75.45	79.80	85.61
Temperature entire pregnancy	**Control**	11.17	15.44	16.59	18.08	19.82	21.09	21.84	24.15	**Control**	10.76	14.07	16.20	18.14	20.24	22.29	23.14	24.51
	**Preterm birth**	12.35	15.42	16.57	18.08	19.84	21.09	21.88	24.01	**Stillbirth**	11.42	14.17	16.15	18.04	20.23	22.32	23.10	24.25
Temperature trimester 1	**Control**	9.65	12.71	14.57	18.22	21.49	23.50	24.42	25.55	**Control**	9.76	12.70	14.55	18.30	21.58	23.48	24.40	25.51
	**Preterm birth**	9.65	12.67	14.60	18.35	21.55	23.55	24.45	25.51	**Stillbirth**	10.25	12.71	14.54	18.18	21.49	23.57	24.49	25.40
Temperature trimester 2	**Control**	9.34	12.72	14.61	18.35	21.60	23.57	24.43	26.56	**Control**	9.07	12.48	14.46	18.38	21.64	23.79	24.94	26.85
	**Preterm birth**	9.71	12.67	14.58	18.36	21.62	23.59	24.44	26.22	**Stillbirth**	9.80	12.37	14.55	18.59	21.82	23.93	24.80	26.09
Temperature trimester 3	**Control**	6.83	12.24	14.41	18.83	21.97	24.22	25.47	32.48	**Control**	8.84	12.26	14.40	18.72	21.98	24.22	25.75	32.45
	**Preterm birth**	8.22	12.16	14.26	18.67	21.89	24.21	25.45	33.64	**Stillbirth**	8.81	12.15	14.36	18.60	22.12	24.21	25.69	31.11

[a] Controls are any births with gestational age > gestational age of case, [b] Controls are any ongoing pregnancies with gestational age > gestational age of case, or live births of at least the same gestational age as a case.

Preterm birth matched set: N for entire pregnancy, trimester 1, trimester 2 = 329,494; N for trimester 3 = 327,758

Exposure and pregnancy window	Preterm birth set [a]	Minimum	5th	25th	Median	75th	95th	99th	Maximum	Stillbirth set [b]	Minimum	5th	25th	Median	75th	95th	99th	Maximum

Stillbirth matched set: N for entire pregnancy, trimester 1 , trimester 2 = 32,208; N for trimester 3 = 30,461

Table S4: Summary statistics of exposure variables for preterm birth and stillbirth matched sets during different pregnancy windows excluding births from the unprecedented bushfires during summer 2019 – 2020 (all births after 31 October 2019 are excluded)

Exposure and pregnancy window	Preterm birth set [a]	Minimum	5th	25th	Median	75th	95th	99th	Maximum	Stillbirth set [b]	Minimum	5th	25th	Median	75th	95th	99th	Maximum
All-source $PM_{2.5}$ entire pregnancy	**Control**	5.13	6.15	6.89	7.52	8.02	8.72	9.17	10.71	**Control**	5.04	6.07	6.87	7.46	8.01	8.82	9.47	10.79
	**Preterm birth**	5.29	6.15	6.89	7.53	8.04	8.75	9.20	10.23	**Stillbirth**	5.10	6.11	6.96	7.53	8.09	8.94	9.56	10.70
All-source $PM_{2.5}$ trimester 1	**Control**	4.74	5.83	6.78	7.35	8.02	9.24	10.06	11.78	**Control**	4.76	5.81	6.77	7.34	8.00	9.23	10.08	11.27
	**Preterm birth**	4.81	5.83	6.77	7.35	8.03	9.28	10.08	11.46	**Stillbirth**	4.94	5.83	6.85	7.39	8.08	9.34	10.01	11.28
All-source $PM_{2.5}$ trimester 2	**Control**	4.69	5.84	6.80	7.41	8.10	9.33	10.11	14.56	**Control**	4.45	5.76	6.72	7.39	8.12	9.47	10.41	12.93
	**Preterm birth**	4.80	5.84	6.80	7.41	8.12	9.37	10.16	11.96	**Stillbirth**	4.84	5.82	6.80	7.46	8.15	9.57	10.61	12.36

Exposure and pregnancy window	Preterm birth set [a]	Minimum	5th	25th	Median	75th	95th	99th	Maximum	Stillbirth set [b]	Minimum	5th	25th	Median	75th	95th	99th	Maximum
All-source $PM_{2.5}$ trimester 3	**Control**	2.83	5.61	6.62	7.42	8.24	9.81	11.02	50.06	**Control**	2.88	5.48	6.59	7.40	8.26	9.89	11.60	38.16
	**Preterm birth**	3.01	5.63	6.62	7.41	8.25	9.85	11.05	20.91	**Stillbirth**	3.44	5.54	6.66	7.40	8.29	10.19	11.78	48.84
Biomass - attributable $PM_{2.5}$ entire pregnancy	**Control**	0.00	0.00	0.06	0.17	0.39	0.67	0.90	2.62	**Control**	0.00	0.00	0.05	0.14	0.36	0.76	1.08	2.47
	**Preterm birth**	0.00	0.00	0.06	0.17	0.40	0.70	0.95	1.84	**Stillbirth**	0.00	0.00	0.05	0.15	0.38	0.79	1.10	1.84
Biomass - attributable $PM_{2.5}$ trimester 1	**Control**	0.00	0.00	0.00	0.10	0.28	1.00	1.50	3.66	**Control**	0.00	0.00	0.00	0.09	0.27	0.99	1.52	3.66
	**Preterm birth**	0.00	0.00	0.00	0.09	0.28	1.03	1.54	3.39	**Stillbirth**	0.00	0.00	0.00	0.10	0.29	1.05	1.47	2.72
Biomass - attributable $PM_{2.5}$ trimester 2	**Control**	0.00	0.00	0.00	0.10	0.32	1.08	1.56	6.02	**Control**	0.00	0.00	0.00	0.09	0.29	1.15	1.89	5.04
	**Preterm birth**	0.00	0.00	0.00	0.10	0.32	1.11	1.62	3.62	**Stillbirth**	0.00	0.00	0.00	0.09	0.30	1.15	1.97	3.67
Biomass - attributable $PM_{2.5}$ trimester 3	**Control**	0.00	0.00	0.00	0.00	0.29	1.31	2.32	42.83	**Control**	0.00	0.00	0.00	0.00	0.26	1.22	2.41	30.76
	**Preterm birth**	0.00	0.00	0.00	0.00	0.29	1.32	2.32	14.07	**Stillbirth**	0.00	0.00	0.00	0.00	0.28	1.35	2.75	41.13
	**Control**	0.00	0.00	2.00	6.00	11.00	19.00	25.00	44.00	**Control**	0.00	0.00	1.00	4.00	9.00	17.00	23.00	44.00

**Exposure and pregnancy window**	**Preterm birth set [a]**	**Minimum**	**5th**	**25th**	**Median**	**75th**	**95th**	**99th**	**Maximum**	**Stillbirth set [b]**	**Minimum**	**5th**	**25th**	**Median**	**75th**	**95th**	**99th**	**Maximum**
Number of biomass events entire pregnancy	**Preterm birth**	0.00	0.00	2.00	6.00	11.00	19.00	26.00	42.00	**Stillbirth**	0.00	0.00	1.00	4.00	10.00	17.00	22.00	29.00
Number of biomass events trimester 1	**Control**	0.00	0.00	0.00	1.00	4.00	11.00	16.00	25.00	**Control**	0.00	0.00	0.00	1.00	4.00	11.00	16.00	24.00
	**Preterm birth**	0.00	0.00	0.00	1.00	4.00	11.00	16.00	23.00	**Stillbirth**	0.00	0.00	0.00	1.00	4.00	11.00	16.00	21.00
Number of biomass events trimester 2	**Control**	0.00	0.00	0.00	1.00	4.00	12.00	16.00	25.00	**Control**	0.00	0.00	0.00	1.00	3.00	10.00	15.00	25.00
	**Preterm birth**	0.00	0.00	0.00	2.00	4.00	12.00	17.00	25.00	**Stillbirth**	0.00	0.00	0.00	1.00	3.00	10.00	15.00	22.00
Number of biomass events trimester 3	**Control**	0.00	0.00	0.00	0.00	2.00	7.00	12.00	20.00	**Control**	0.00	0.00	0.00	0.00	2.00	8.00	13.00	22.00
	**Preterm birth**	0.00	0.00	0.00	0.00	2.00	7.00	12.00	19.00	**Stillbirth**	0.00	0.00	0.00	0.00	2.00	9.00	13.00	17.00

[a] Controls are any births with gestational age > gestational age of case, [b] Controls are any ongoing pregnancies with gestational age > gestational age of case, or live births of at least the same gestational age as a case.

Preterm birth matched set: N for entire pregnancy, trimester 1, trimester 2 = 307,870; N for trimester 3 = 306,204

Stillbirth matched set: N for entire pregnancy, trimester 1 , trimester 2 = 30,248; N for trimester 3 = 28,571

Table S5: Preterm birth spearman correlation for entire pregnancy exposure

Variable	Biomass attributable $PM_{2.5}$ entire pregnancy	All-source $PM_{2.5}$ entire pregnancy	Temperature entire pregnancy	Relative humidity entire pregnancy
Biomass attributable $PM_{2.5}$ entire pregnancy	1,00	0,80	-0,05	-0,29
All-source $PM_{2.5}$ entire pregnancy		1,00	0,05	-0,31
Temperature entire pregnancy			1,00	-0,08
Relative humidity entire pregnancy				1,00

Note: p-values for all < .001, Preterm birth matched set: N for entire pregnancy = 329,494

Table S6: Preterm birth spearman correlation for first trimester exposure

Variable	Biomass attributable $PM_{2.5}$ trimester 1	All-source $PM_{2.5}$ trimester 1	Temperature trimester 1	Relative humidity trimester 1
Biomass attributable $PM_{2.5}$ trimester 1	1,00	0,69	-0,29	-0,24
All-source $PM_{2.5}$ trimester 1		1,00	-0,13	-0,24
Temperature trimester 1			1,00	0,02
Relative humidity trimester 1				1,00

Note: p-values for all < .001, Preterm birth matched set: N for trimester 1 = 329,494

Table S7: Preterm birth spearman correlation for second trimester exposure

Variable	Biomass attributable $PM_{2.5}$ trimester 2	All-source $PM_{2.5}$ trimester 2	Temperature trimester 2	Relative humidity trimester 2
Biomass attributable $PM_{2.5}$ trimester 2	1,00	0,73	-0,23	-0,28
All-source $PM_{2.5}$ trimester 2		1,00	-0,07	-0,27
Temperature trimester 2			1,00	-0,02
Relative humidity trimester 2				1,00

Note: p-values for all < .001, Preterm birth matched set: N for trimester 2 = 329,494

Table S8: Preterm birth spearman correlation for third trimester exposure

Variable	Biomass attributable $PM_{2.5}$ trimester 3	All-source $PM_{2.5}$ trimester 3	Temperature trimester 3	Relative humidity trimester 3
Biomass attributable $PM_{2.5}$ trimester 3	1,00	0,68	-0,21	-0,22
All-source $PM_{2.5}$ trimester 3		1,00	-0,06	-0,26
Temperature trimester 3			1,00	0,01
Relative humidity trimester 3				1,00

Note: p-values for all < .001, Preterm birth matched set: N for trimester 3 = 327,758

Table S9: Stillbirth spearman correlation for entire pregnancy exposure

Variable	Biomass attributable $PM_{2.5}$ entire pregnancy	All-source $PM_{2.5}$ entire pregnancy	Temperature entire pregnancy	Relative humidity entire pregnancy
Biomass attributable $PM_{2.5}$ entire pregnancy	1,00	0,77	-0,19	-0,28
All-source $PM_{2.5}$ entire pregnancy		1,00	-0,05	-0,30
Temperature entire pregnancy			1,00	0,01
Relative humidity entire pregnancy				1,00

Note: p-values for all < .001, Stillbirth matched set: N for entire pregnancy = 32,208

Table S10: Stillbirth spearman correlation for entire pregnancy exposure

Variable	Biomass attributable $PM_{2.5}$ trimester 1	All-source $PM_{2.5}$ trimester 1	Temperature trimester 1	Relative humidity trimester 1
Biomass attributable $PM_{2.5}$ trimester 1	1,00	0,69	-0,29	-0,23
All-source $PM_{2.5}$ trimester 1		1,00	-0,13	-0,23
Temperature trimester 1			1,00	0,03
Relative humidity trimester 1				1,00

Note: p-values for all < .001, Stillbirth matched set: N for trimester 1 = 32,208

Table S11: Stillbirth spearman correlation for entire pregnancy exposure

Variable	Biomass attributable $PM_{2.5}$ trimester 2	All-source $PM_{2.5}$ trimester 2	Temperature trimester 2	Relative humidity trimester 2
Biomass attributable $PM_{2.5}$ trimester 2	1,00	0,70	-0,23	-0,26
All-source $PM_{2.5}$ trimester 2		1,00	-0,07	-0,24
Temperature trimester 2			1,00	-0,05
Relative humidity trimester 2				1,00

Note: p-values for all < .001, Stillbirth matched set: N for trimester 2 = 32,208

Table S12: Stillbirth spearman correlation for entire pregnancy exposure

Variable	Biomass attributable $PM_{2.5}$ trimester 3	All-source $PM_{2.5}$ trimester 3	Temperature trimester 3	Relative humidity trimester 3
Biomass attributable $PM_{2.5}$ trimester 3	1,00	0,64	-0,17	-0,21
All-source $PM_{2.5}$ trimester 3		1,00	-0,02	-0,22
Temperature trimester 3			1,00	0,00
Relative humidity trimester 3				1,00

Note: p-values for all < .001, Stillbirth matched set: N for trimester 3 = 30,461

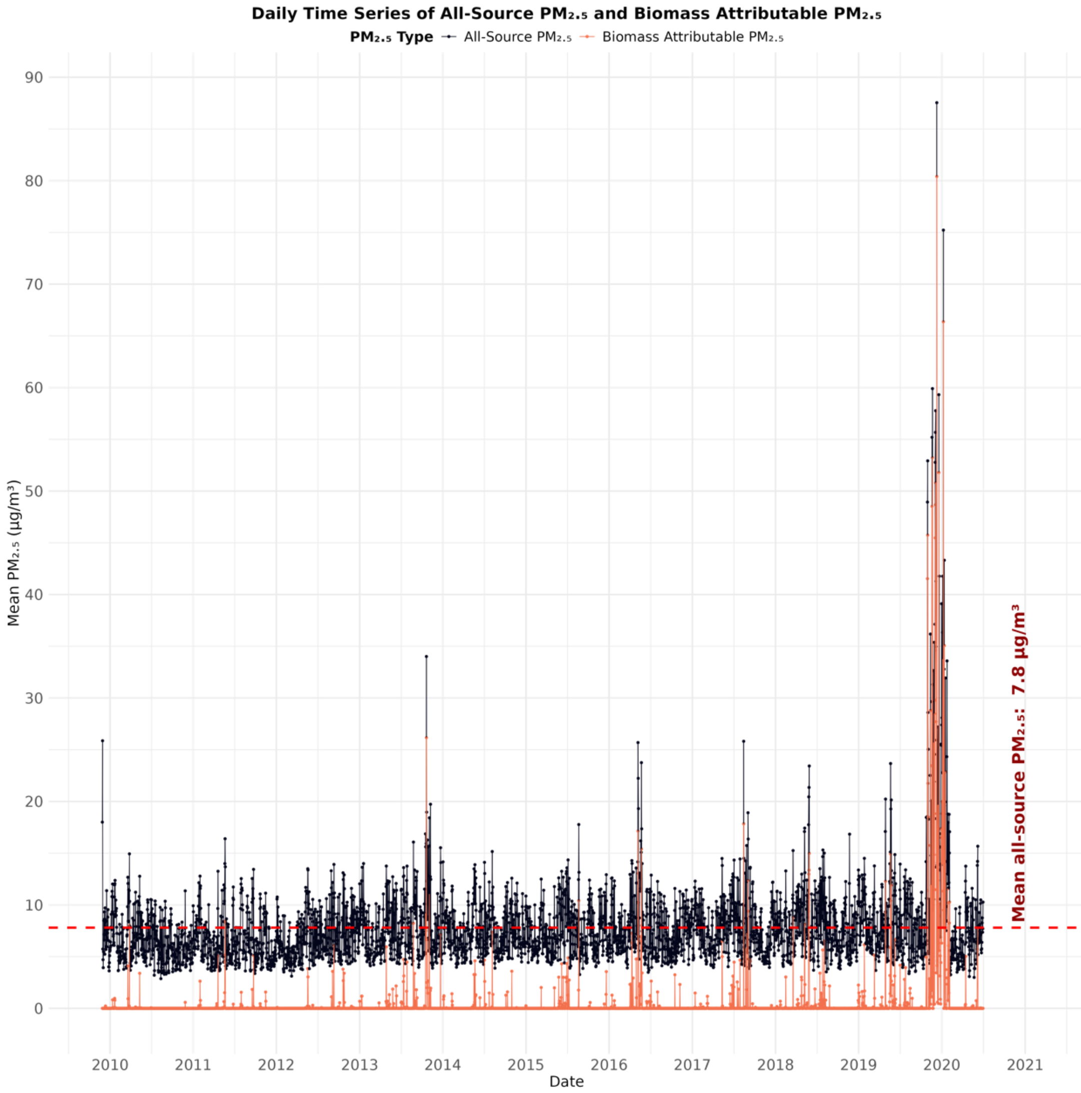

Figure S3: Daily time series (28 November 2009 – 30 June 2020) averaged over greater metropolitan Sydney for all-source $PM_{2.5}$ and biomass-attributable $PM_{2.5}$.

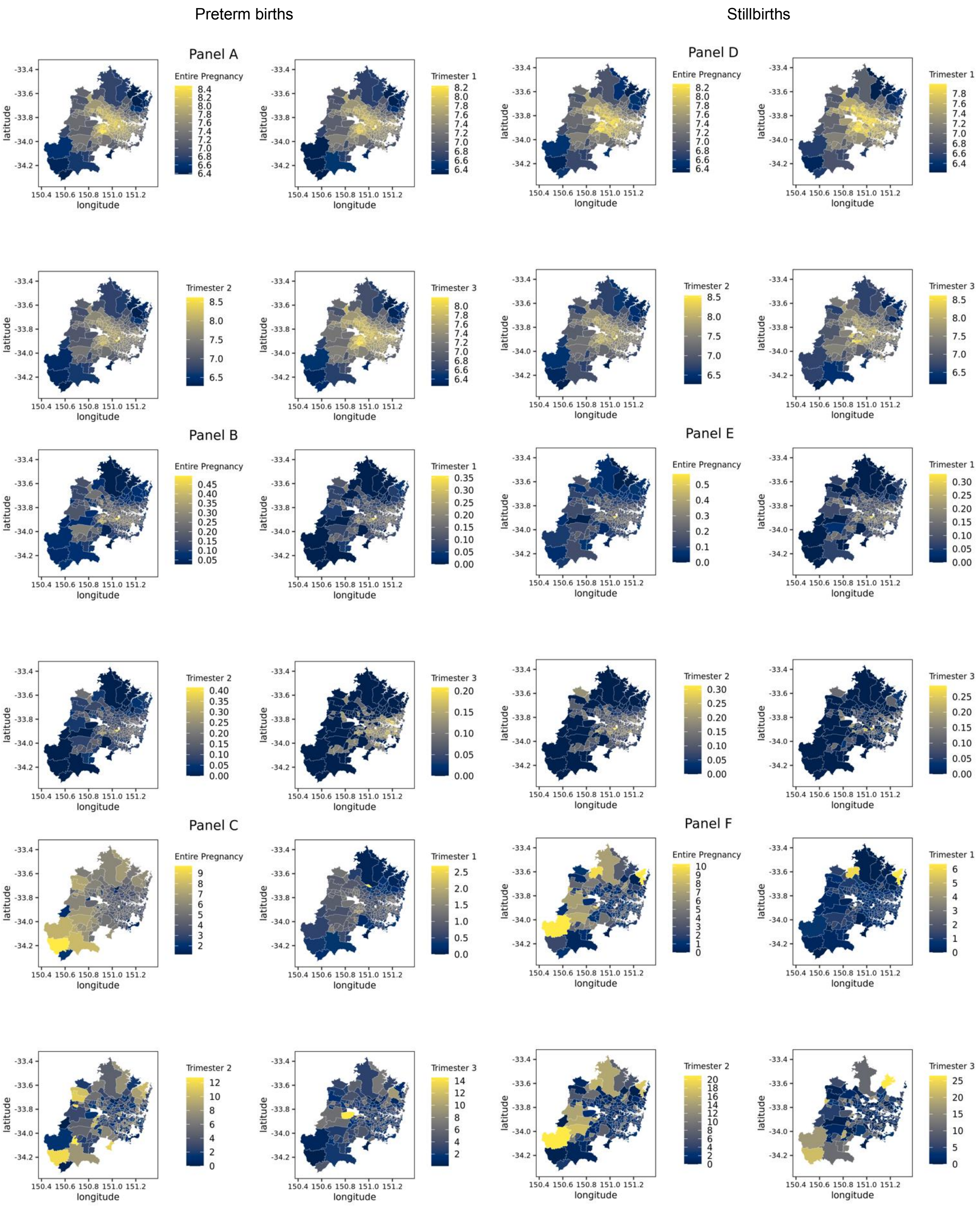

Figure S4: Spatial distribution of median all-source (Panel A and Panel D) and biomass-attributable $PM_{2.5}$ (Panel B and Panel E) across different pregnancy windows in metropolitan Sydney for births born between 24 April 2010 – 23 June 2020 aggregated over a statistical area level 2. Panel C and F show births after 15 October 2019 to show biomass-attributable $PM_{2.5}$ exposure for the extreme wildfires between late 2019 and early 2020.

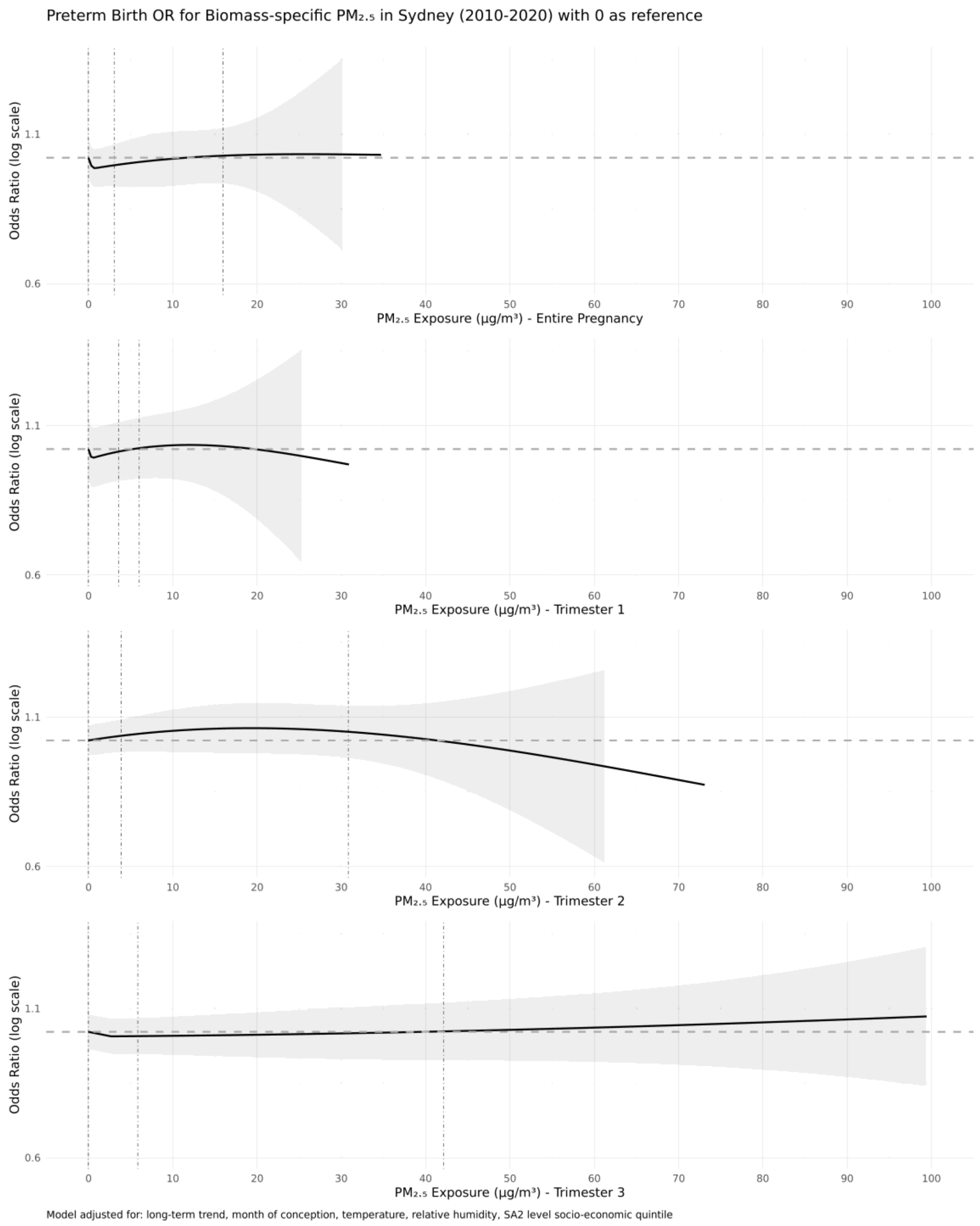

Figure S5: Exposure-response relationship between biomass-attributable $PM_{2.5}$ and preterm births for biomass-attributable $PM_{2.5}$ modelled as a natural cubic spline with 3 degrees of freedom for different pregnancy windows

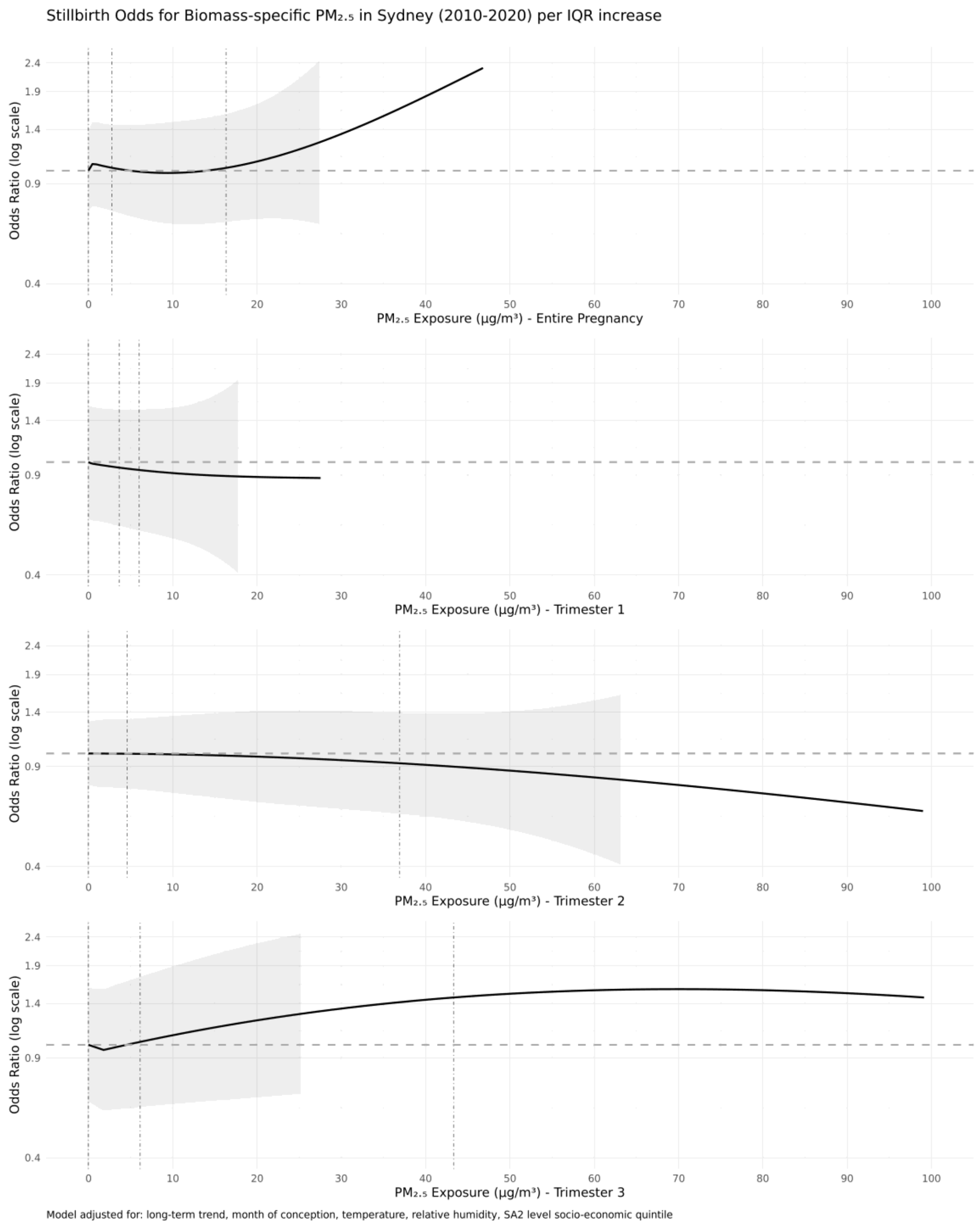

Figure S6: Exposure-response relationship between biomass-attributable $PM_{2.5}$ and stillbirths for biomass-attributable $PM_{2.5}$ modelled as a natural cubic spline with 3 degrees of freedom for different pregnancy windows. $PM_{2.5}$ = 0 is the reference

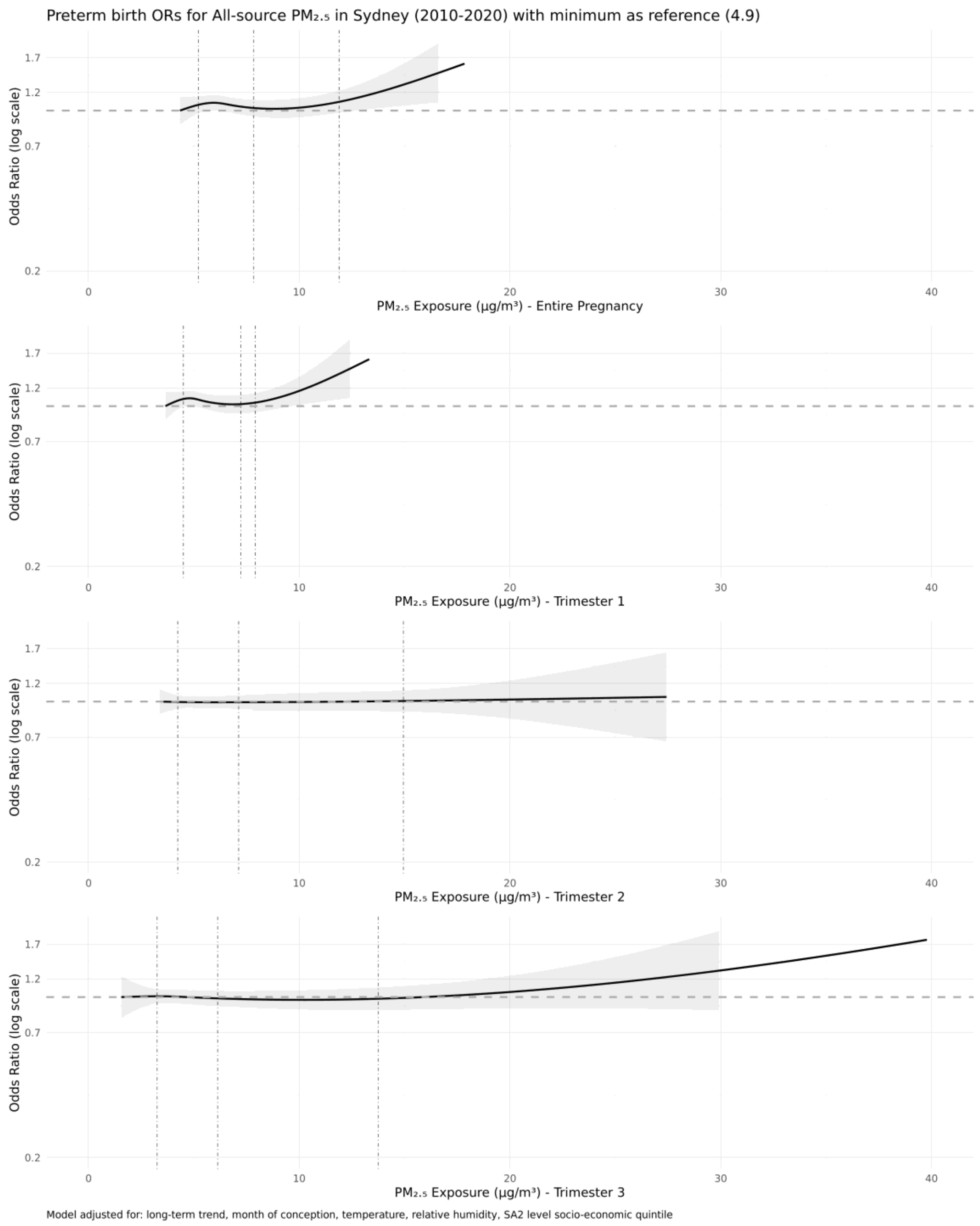

Figure S7: Exposure-response relationship between all-source $PM_{2.5}$ and preterm births for all-source $PM_{2.5}$ modelled as a natural cubic spline with 3 degrees of freedom for different pregnancy windows

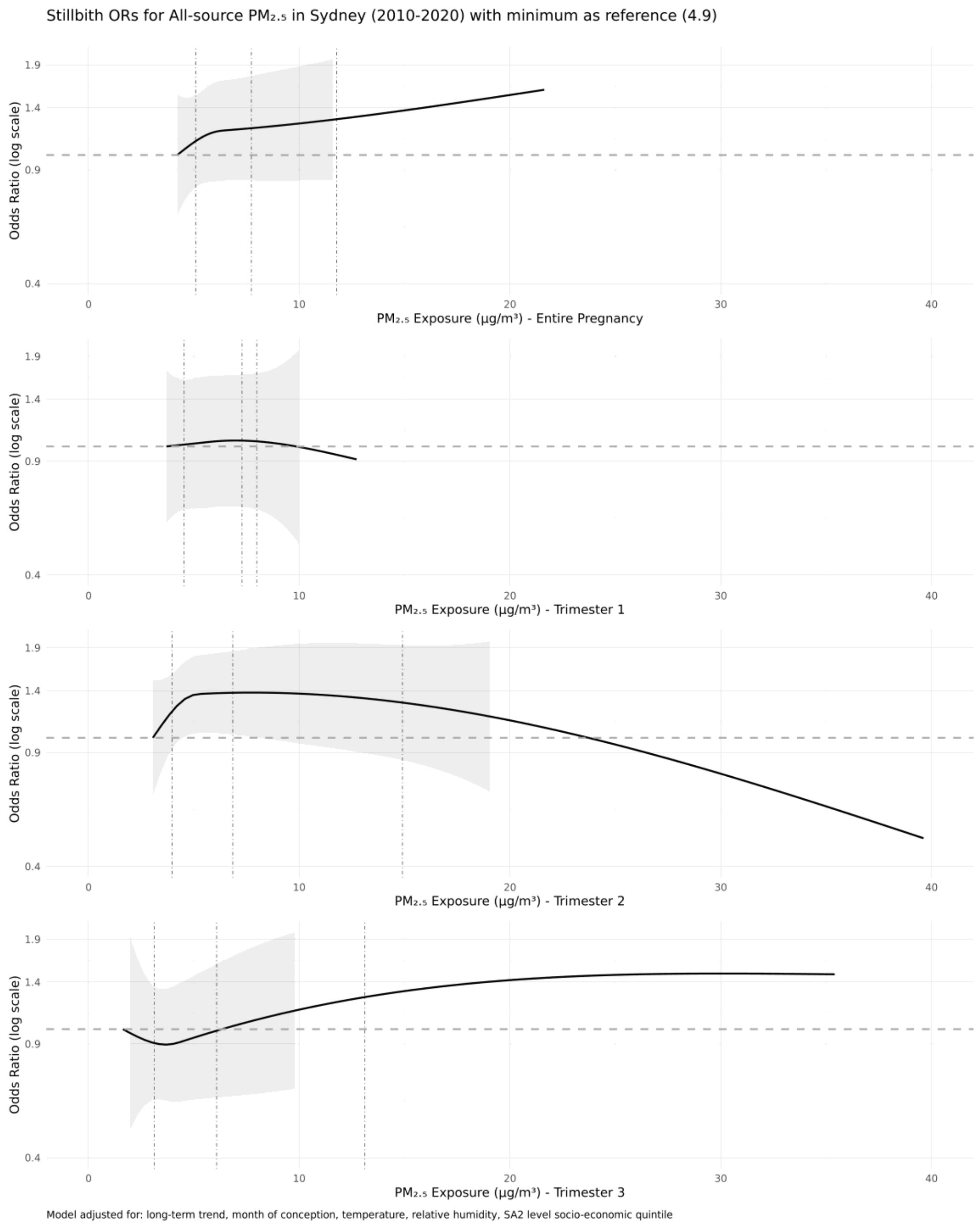

Figure S8: Exposure-response relationship between all-source $PM_{2.5}$ and stillbirths for all-source $PM_{2.5}$ modelled as a natural cubic spline with 3 degrees of freedom for different pregnancy windows

Table S13: Preterm birth and stillbirth odds ratio for exposure to biomass-attributable $PM_{2.5}$ and all-source $PM_{2.5}$ across different pregnancy exposure windows in unadjusted models

	**Biomass-attributable $PM_{2.5}$**		**All-source $PM_{2.5}$**	
**Model**	**Unadjusted OR**	**IQR (µg/m³)**	**Unadjusted OR**	**IQR (µg/m³)**
Preterm birth - entire pregnancy	1.000 (0.996, 1.004)	0.37	1.001 (0.991, 1.011)	1.18
Preterm birth - trimester 1	0.999 (0.991, 1.006)	0.32	0.993 (0.979, 1.008)	1.30
Preterm birth - trimester 2	1.000 (0.997, 1.002)	0.38	1.000 (0.993, 1.007)	1.38
Preterm birth - trimester 3	1.000 (0.998, 1.002)	0.34	1.001 (0.993, 1.008)	1.72
Stillbirth - entire pregnancy	1.011 (0.998, 1.025)	0.37	1.053 (1.021, 1.086)	1.20
Stillbirth - trimester 1	1.013 (0.99, 1.037)	0.31	1.064 (1.017, 1.113)	1.29
Stillbirth - trimester 2	1.003 (0.997, 1.01)	0.33	1.023 (1.002, 1.044)	1.45
Stillbirth - trimester 3	1.004 (0.997, 1.011)	0.31	1.024 (0.993, 1.057)	1.76

Note: Odds ratios (ORs) were estimated using conditional logistic regression models. Unadjusted model with only biomass-attributable $PM_{2.5}$ (µg/m³) or all-source $PM_{2.5}$ (µg/m³) included. ORs are expressed per interquartile range (IQR) increase in exposure. Units are micrograms per cubic meter (µg/m³).

Table S14: Preterm birth and stillbirth odds ratio for exposure to biomass-attributable $PM_{2.5}$ and all-source $PM_{2.5}$ across different pregnancy exposure windows for sensitivity analyses in which a) all confounders were included and b) only the trend component from daily predicted $PM_{2.5}$ values were removed

	**Model with all confounders included [a]**				**Model with only trend decomposition removed [b]**	
	**Biomass-attributable $PM_{2.5}$**		**All-source $PM_{2.5}$**		**Biomass-attributable $PM_{2.5}$**	
**Model**	**Adjusted OR**	**IQR (μg/m³)**	**Adjusted OR**	**IQR (μg/m³)**	**Adjusted OR**	**IQR (μg/m³)**
Preterm birth - entire pregnancy	1.002 (0.997, 1.007)	0.37	1.002 (0.988, 1.016)	1.18	1.002 (0.997, 1.008)	0.39
Preterm birth - trimester 1	1.001 (0.992, 1.009)	0.32	0.983 (0.964, 1.002)	1.30	1.001 (0.991, 1.010)	0.36
Preterm birth - trimester 2	1.001 (0.998, 1.004)	0.38	1.002 (0.993, 1.010)	1.38	1.001 (0.998, 1.004)	0.42
Preterm birth - trimester 3	1.000 (0.998, 1.002)	0.34	1.000 (0.991, 1.008)	1.72	1.000 (0.998, 1.002)	0.36
Stillbirth - entire pregnancy	1.002 (0.985, 1.019)	0.37	1.023 (0.979, 1.070)	1.20	1.002 (0.983, 1.020)	0.40
Stillbirth - trimester 1	0.991 (0.962, 1.021)	0.31	1.010 (0.947, 1.076)	1.29	0.990 (0.958, 1.022)	0.35
Stillbirth - trimester 2	0.998 (0.990, 1.005)	0.33	0.999 (0.972, 1.027)	1.45	0.997 (0.989, 1.005)	0.37
Stillbirth - trimester 3	1.006 (0.999, 1.014)	0.31	1.031 (0.992, 1.070)	1.76	1.007 (0.999, 1.015)	0.34

Note: Odds ratios (OR) were estimated using conditional logistic regression models. ORs are expressed per interquartile range (IQR) increase in exposure. Units are micrograms per cubic meter (μg/m³). Controls were matched randomly to cases. Gestational age of controls was truncated to the same age as their matched case.

[a] Models adjusted for long-term-trend, month of conception, temperature, relative humidity, and statistical area 2 level socio-economic quintile, mother's age, parity, mother's smoking status, sex of infant, non-biomass-attributable $PM_{2.5}$ (μg/m³).

[b] Models adjusted for long-term-trend, month of conception, temperature, relative humidity, and statistical area 2 level socio-economic quintile.

Table S15: Preterm birth and stillbirth odds ratio for exposure to biomass-attributable $PM_{2.5}$ and all-source $PM_{2.5}$ across different pregnancy exposure windows for sensitivity analyses including only births before November 2019

	**Biomass-attributable $PM_{2.5}$**		**All-source $PM_{2.5}$**	
**Model**	**Adjusted OR**	**IQR (µg/m³)**	**Adjusted OR**	**IQR (µg/m³)**
Preterm birth - entire pregnancy	1.002 (0.977, 1.027)	0.32	0.995 (0.963, 1.028)	1.14
Preterm birth - trimester 1	0.997 (0.985, 1.010)	0.27	0.982 (0.949, 1.015)	1.24
Preterm birth - trimester 2	1.009 (0.995, 1.023)	0.31	0.996 (0.974, 1.019)	1.29
Preterm birth - trimester 3	0.993 (0.985, 1.002)	0.29	0.997 (0.962, 1.033)	1.63
Stillbirth - entire pregnancy	0.979 (0.920, 1.039)	0.30	1.003 (0.920, 1.087	1.12
Stillbirth - trimester 1	0.980 (0.923, 1.037)	0.27	0.971 (0.905, 1.037)	1.22
Stillbirth - trimester 2	0.989 (0.954, 1.025)	0.29	1.008 (0.940, 1.077)	1.35
Stillbirth - trimester 3	1.012 (0.991, 1.034)	0.26	1.030 (0.952, 1.115)	1.63

Note: Odds ratios (ORs) were estimated using conditional logistic regression models. Unadjusted model with only biomass-attributable $PM_{2.5}$ (µg/m³) or all-source $PM_{2.5}$ (µg/m³) included. ORs are expressed per interquartile range (IQR) increase in exposure. Units are micrograms per cubic meter (µg/m³).

Preterm birth matched set: N for entire pregnancy, trimester 1, trimester 2 = 307,870; N for trimester 3 = 306,204

Stillbirth matched set: N for entire pregnancy, trimester 1 , trimester 2 = 30,248; N for trimester 3 = 28,571

Table S16: Preterm birth and stillbirth hazard ratios. for exposure to biomass-attributable $PM_{2.5}$ across different pregnancy exposure windows in Cox proportional hazard model as a sensitivity analysis

	**Biomass-attributable $PM_{2.5}$**		**All-source $PM_{2.5}$**	
**Model**	**Adjusted HR**	**IQR (μg/m³)**	**Adjusted HR**	**IQR (μg/m³)**
Preterm birth - entire pregnancy	1.008 (0.995, 1.022)	0.37	1.002 (0.991, 1.014)	1.18
Stillbirth - entire pregnancy	1.000 (0.983, 1.017)	0.37	1.017 (0.970, 1.067)	1.20

Note: Hazard ratios (HRs) were estimated using time varying Cox proportional hazard models, adjusted for long-term trend, month of conception, temperature, relative humidity, statistical area 2 level socio-economic quintile. HRs are expressed per interquartile range (IQR) increase in exposure. Units are micrograms per cubic meter (μg/m³). The proportional hazards assumption was tested using Schoenfeld residuals and was initially violated for both biomass-attributable and all-source $PM_{2.5}$ exposure in the Cox regression analyses for preterm births and stillbirths. To address this violation, we stratified the models by statistical area 2 level socio-economic quintile, which resolved the proportionality issues while maintaining stable effect estimates. For instance, the HR for biomass-attributable $PM_{2.5}$ exposure and preterm birth changed minimally from 1.008 (95% CI: 0.995-1.022) in the original model to 1.008 (95% CI: 0.994-1.022) in the stratified analysis, demonstrating robustness of the primary association to this adjustment. Similar stability in estimates was observed for stillbirth analyses.
